# A degron system targeting endogenous PD-1 inhibits the growth of tumor cells in mice

**DOI:** 10.1093/narcan/zcac019

**Published:** 2022-06-17

**Authors:** Chie Naruse, Kazushi Sugihara, Tatsuhiko Miyazaki, Xuchi Pan, Fumihiro Sugiyama, Masahide Asano

**Affiliations:** Institute of Laboratory Animals, Graduate School of Medicine, Kyoto University, Yoshida-Konoe-cho, Sakyo-ku, Kyoto 606-8501, Japan; Institute of Laboratory Animals, Graduate School of Medicine, Kyoto University, Yoshida-Konoe-cho, Sakyo-ku, Kyoto 606-8501, Japan; Department of Pathology, Gifu University Hospital, 1-1 Yanagido, Gifu 501-1104, Japan; Institute of Laboratory Animals, Graduate School of Medicine, Kyoto University, Yoshida-Konoe-cho, Sakyo-ku, Kyoto 606-8501, Japan; Laboratory Animal Resource Center, Transborder Medical Research Center, Faculty of Medicine, University of Tsukuba, 1-1-1 Tennodai, Tsukuba, Ibaraki 305-8575, Japan; Institute of Laboratory Animals, Graduate School of Medicine, Kyoto University, Yoshida-Konoe-cho, Sakyo-ku, Kyoto 606-8501, Japan

## Abstract

Recently, targeted protein degradation systems have been developed using the ubiquitin-proteasome system. Here, we established Programmed cell death-1 (PD-1) knockdown mice as a model system for subjecting endogenous mouse proteins to the small molecule-assisted shutoff (SMASh) degron system. SMASh degron-tagged PD-1-mCherry in Jurkat cells and CD3^+^ splenocytes were degraded by the NS3/4A protease inhibitors, asunaprevir (ASV) or grazoprevir (GRV). Growth of MC-38 colon adenocarcinoma cells injected in *Pdcd1-mCherry-SMASh* homozygous knock-in (KI) mice was repressed by ASV or GRV. Moreover, growth of MC-38 cells was suppressed in wild-type mice transplanted with KI bone marrow cells after GRV treatment. This is the first study to use a degron tag targeting an endogenous mouse protein *in vivo*. Our experimental system using the SMASh degron may be employed for treating diseases and characterizing the cellular functions of essential proteins.

## Graphical Abstract

**Graphical Abstract:**
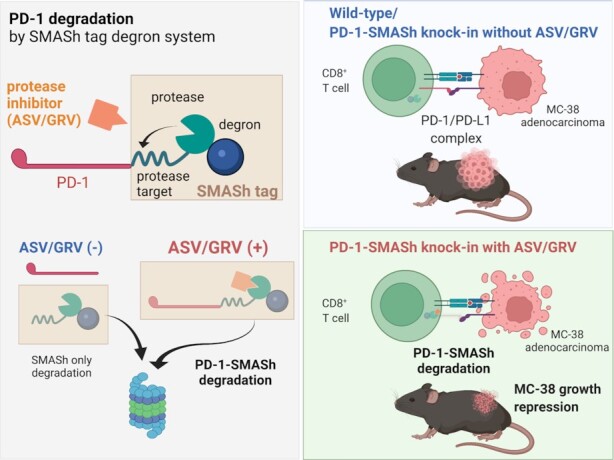
We applied the SMASh degron to a cancer immunotherapy model to reduce cancerous growth. This system may be employed for characterizing the cellular functions of proteins and treating diseases.

## INTRODUCTION

Recently, targeted protein degradation systems have been developed using the ubiquitin-proteasome system. In this system, the E3 ubiquitin ligases recognize degron and target proteins are ubiquitinated. In turn, the ubiquitinated proteins are degraded by the proteasomes to approximately 10 amino acids long peptides. In the targeted protein degradation system, the protein-of-interest is ubiquitinated and degraded rapidly upon addition of small molecule compounds. After removal of the compounds, the target protein is allowed to re-express. Unlike gene knockout, protein removal is reversible. Since mRNA is not removed, protein recovery after drug removal is rapid. Various degron systems, such as the auxin-inducible degron (AID) system (1,2), FKBP-based systems (3–5), degron systems with HaloPROTAC tag (6–8), and IMiD-induced degron systems, such as IKZF (9) and SALL4 (10), have been used to elucid ate the functions of various proteins in cultured cells (11–14). Degron tags have also been used in nematodes (15) and zebrafish (16,17) under *in vivo* conditions. Studies have demonstrated that the expression of transgene-derived proteins fused to degron tags in cancer cell lines and T cells implanted in mice was reduced following drug administration (9,18,19). The AID2 system, which is a modification of the AID system, can be used *in vivo* in mice because the degradation-promoting agent is more specific and less toxic when compared with the conventional AID system (20). However, the AID and AID2 systems require the introduction of an exogenous TIR E3 ubiquitin ligase in addition to the AID tag, which is not easy to introduce into the body. So far, the use of degron tags in mice has been limited to exogenous marker proteins, and there are no examples of their use for endogenous proteins in mice.

In the present study, we focused on the small molecule-assisted shutoff (SMASh) degron tag containing the NS3 protease domain, the cleavage sequence for NS3 and NS4A from human hepatitis C virus (HCV). The SMASh system has certain advantages, i.e., the tag consists of a single component, the length of the tag is optimal for genome editing, and the drugs used are approved for humans (21–25). NS4A contains a hydrophobic amino acid that lacks a membrane localization signal and exhibits degron-like activity. The SMASh tag bound to protein-of-interest (POI) is usually detached from POI by NS3 protease, resulting in stable expression of POI. The addition of asunaprevir (ASV), a drug that inhibits NS3 protease activity, stabilizes the bond between POI and SMASh tag, resulting in the degradation of POI with NS4A. Considering such a mechanism, when the SMASh tag is used, the POI synthesized after the addition of ASV is degraded within a few hours, but the degradation time of the POI synthesized before the addition of ASV is considered the original degradation time.

Research on novel treatment approaches for cancer, such as cancer immunotherapy, has advanced substantially (26–28). In cancer immunotherapy, antibodies that interfere with the functions of immune checkpoint molecules such as Programmed cell death-1 (PD-1, encoded by *Pdcd1*) and Cytotoxic T-lymphocyte-associated protein 4 (CTLA-4) are administered to cancer patients. However, suppression of the functions of immune checkpoint molecules is disadvantageous for the long-term health of patients because they play an important role in preventing the occurrence of autoimmune diseases (29–32). Anti-PD-1 antibodies exhibit less severe adverse effects than anti-CTLA-4 antibodies (33,34). However, 71% of the patients develop varying grades of immune-related adverse events upon anti-PD-1 antibody administration (35). The incidence of grade 3 to grade 5 immune-related adverse events associated with anti-PD-1 antibody treatment is approximately 13%, and that associated with a combinatorial therapy of anti-PD-1 antibodies and anticancer drugs is 68% (32). Moreover, some patients do not respond to checkpoint inhibitors such as anti-PD1 antibodies; therefore, further research on the mechanisms of PD1-mediated immunomodulation is required.

In the present study, we developed a system that degrades endogenous PD-1 protein only for the minimum period necessary to eliminate cancer cells. By reversibly knocking down PD-1 for a short period using the SMASh degron system, we attempted to reduce cancerous growth and minimize the risk of autoimmune diseases. This is the first study to use the SMASh degron tag in mice and demonstrate its potential utility in the temporal control of endogenous protein expression.

## MATERIALS AND METHODS

### Animal experiments

All mice were maintained under specific pathogen-free conditions at the Institute of Laboratory Animals, Kyoto University Graduate School of Medicine. The mice were maintained at 24 ± 2°C and 60 ± 5% humidity under a 14-h light (07:00−21:00)/dark cycle. The mice were fed with F-2 (Funabashi Farm, Chiba) and water. All animal experiments were conducted according to the Fundamental Guidelines for the Proper Conduct of Animal Experiment and Related Activities in Academic Research Institutions under the jurisdiction of the Ministry of Education, Culture, Sports, Science and Technology of Japan, and were approved by the Animal Experimentation Committee of Kyoto University (approval numbers: MedKyo19554, 20006 and 21003). Experiments were performed in a non-blinded condition.

### DNA oligos and DNA sequences

All DNA oligos shown in Supplementary Table S2 were synthesized by Fasmac Co., Ltd. (Kanagawa, Japan). Sanger sequencing was performed by Macrogen Japan (Tokyo, Japan) and Medical Research Support Center, Graduate School of Medicine, Kyoto University.

### Preparation and culture conditions of mCherry-SMASh-expressing EL4 cells

EL4 murine T-lymphoma cells (ECACC 85023105) were cultured in Dulbecco's modified Eagle's medium (DMEM) supplemented with 10% fetal bovine serum (FBS), GlutaMAX I (Thermo Fisher Scientific, MA), and 100 U/ml of penicillin and streptomycin (Thermo Fisher Scientific). The guide sequence for *ROSA26* (36) was inserted by annealing oligo DNA fragments #1, ligated with pX330-U6-Chimeric_BB-CBh-hSpCas9 (37) (Addgene #42230), and digested with BbsI to produce pX330-ROSA26. For the construction of the p*EF1α-mCherry-SMASh-T2A-neo-pA* KI vector for integration into the *ROSA26* site, the *SMASh* fragment from p*CS6-SMASh-YFP* (21) (Addgene #68852) and the *neo* fragment from p*PGKneobpA* (38) (Addgene #13442) were synthesized using KOD-plus-Neo (TOYOBO, Osaka, Japan) with primers #2 and #3. The *T2A* peptide coding fragment was generated by annealing oligo DNA fragments #4 and amplifying them with KOD-plus-Neo. These fragments were purified using the NucleoSpin Gel and PCR Clean-up kit (Takara Bio, Shiga, Japan) and assembled with p*EF1α-mCherry-C1* (Takara Bio) digested with EcoRI and KpnI using the NEBuilder HiFi DNA Assembly Master Mix (New England Biolab, MA). The sequences of the coding region were confirmed by Sanger sequencing. The plasmids p*X330-ROSA26* and p*EF1α-mCherry-SMASh-T2A-neo-pA* were transfected into EL4 cells using the Neon Transfection System (Thermo Fisher Scientific) at 1080 V, 50 ms, 1 pulse, according to the manufacturer's instruction. Transfected cells were selected by a 2-week selection on geneticin (1 mg/ml; Thermo Fisher Scientific). Integration of the vector into the *ROSA26* locus was confirmed by PCR using primers #5 for 5′ homologous recombination and #6 for 3′ homologous recombination with genomic DNA as the template. For degradation of mCherry, ASV—dissolved in dimethyl sulfoxide (DMSO) at a concentration of 10 mM—was added to the culture medium at a final concentration of 10 μM (1/1000 dilution). For the negative control, the same amount of the vehicle (DMSO) was added to the medium. The signals of mCherry were observed using the BZ-X700 microscope (Keyence, Osaka, Japan).

### Preparation and culture of Jurkat cells that express PD-1-mCherry-SMASh

Jurkat human T-lymphoma cells (TKG0209) were cultured in Roswell Park Memorial Institute (RPMI)-1640 supplemented with 10% FBS, GlutaMax, penicillin-streptomycin and sodium pyruvate. For the construction of p*EF1α-PD-1-mCherry-SMASh-pA* and p*EF1α-mCherry-SMASh-pA*, a SalI site was introduced downstream of *EF1*α, and *mCherry* was removed from p*EF1α-mCherry-SMASh-T2A-neo-pA* using the KOD-Plus Mutagenesis Kit (TOYOBO) with #7 primers. Next, the murine *Pdcd1* cDNA was amplified from the complementary DNA of C57BL/6J mouse using KOD-plus-Neo and #8 primers and inserted into the *Sal*I site upstream of mCherry in p*EF1α-mCherry-SMASh-2A-Neo-pA* using NEBuilder HiFi DNA Assembly Master Mix to create p*EF1α-PD-1-SMASh-T2A-Neo-pA*. Next, p*EF1α-PD-1-SMASh-T2A-Neo-pA* was used as a template to synthesize p*EF1α-PD-1-pA* using primers #9. A *Sca*I site was introduced between *Pdcd1* and *pA* using primers #10. The *mCherry*-*SMASh* fragment, synthesized by PCR using primers #11 and p*EF1α-mCherry-SMASh-T2A-neo-pA* as a template, was inserted into the *Sca*I site of p*EF1α-PD-1-pA* to produce p*EF1α-PD-1-mCherry-SMASh-pA*. Linker sequences described in Figure 7 were inserted between mCherry and SMASh tag using the KOD-Plus Mutagenesis kit (TOYOBO). The sequences of the coding regions of the vector were confirmed by Sanger sequencing. These plasmids were transfected into Jurkat cells using the Neon Transfection System, following the instructions, at 1325 V, 10 ms, 3 times. Degradation of PD-1 and mCherry was examined immediately after electroporation by adding ASV to the culture medium at a concentration of 10 μM.

### Western blotting

Western blotting was performed as described previously (39). Briefly, cells were lysed with NP-40 lysis buffer (150 mM NaCl, 1% NP-40, 50 mM Tris–Cl pH 8.0) with Complete Protease Inhibitor Cocktail (Sigma-Aldrich, MO), and sonicated using a Bioruptor (SonicBio, Kanagawa, Japan) at high power, on a 30 s-off 30 s-on cycle, 4 times. SDS-PAGE was performed using a 5–12% polyacrylamide gel (FUJIFILM Wako, Osaka, Japan) and NuPAGE MOPS SDS running buffer (Thermo Fisher Scientific) for 1 h, 120 V. One microgram of protein was loaded into each lane. After electrophoresis, proteins were transferred to Immobilon PVDF membranes (Merck, Darmstadt, Germany) in transfer buffer (25 mM Tris, 192 mM glycine, 20% methanol) using Transblot SD cell (Bio-Rad, CA). After blocking the membranes with SuperBlock Blocking Buffer (PBS) (Thermo Fisher Scientific) for 30 min, they were soaked in Can Get Signal 1 with primary antibodies and incubated overnight at 4°C. After three washes with PBST (137 mM NaCl, 2.7 mM KCl, 10 mM Na_2_HPO_4_, 2 mM KH_2_PO_4_, 0.1% Tween 20), the membranes were incubated with secondary antibodies prepared in Can Get Signal 2 (TOYOBO) for 1 h at 20–25°C. After a triple wash with PBST, the signals were detected using Immunostar LD (Fujifilm Wako, Osaka, Japan) and ChemiStage CC-16 Mini (KURABO, Osaka, Japan). Antibodies are shown in Supplementary Table S3.

### Flow cytometry of cultured cells and tumor-infiltrating cells

EL4 and Jurkat cells were harvested and washed twice with fluorescence-activated cell sorting (FACS) solution (2% FBS and 2 mM EDTA in PBS (137 mM NaCl, 2.7 mM KCl, 10 mM Na_2_HPO_4_, 2 mM KH_2_PO_4_)). Tumor-infiltrating cells, which were contained in the MC-38 cell mass, were collected from euthanized mice, along with MC-38 cells. Tumor cell masses were washed with Hanks’ Balanced Salt Solution (HBSS, Thermo Fisher Scientific) and chopped with sterile scissors in Accutase (Nacalai Tesque, Kyoto, Japan) to ensure a size no larger than 3 mm in diameter. All the chopped cell clumps were collected in plastic tubes, and Accutase was added to make a total of 5 ml/g of cell clumps. The tubes were agitated at 37°C for 30 min at 60 rotations per min. Then, the cells were collected through a 70 μm filter. The numbers of cells were counted and adjusted to a density of 1 × 10^7^/ml with FACS solution (PBS containing 2% FBS and 2 mM EDTA). After incubation with Fc blocker, cells were immunostained with antibodies shown in Supplementary Tables S3 and S4. After immunostaining, cells were washed and resuspended in FACS solution with 1/1000 of SYTOX Red dead cell stain (Thermo Fisher Scientific). Cells were analyzed using FACSAria IIIu (Becton, Dickinson and Company, NJ).

### Reverse transcription and quantitative PCR

Total RNA from cells was collected using RNeasy Plus Micro Kit (QIAGEN, Venlo, Netherlands) according to the manufacturer's instruction and the concentration of total RNA was measured using a Q5000 (Tomy Digital Biology, Tokyo, Japan). Total RNA (0.5 μg) was used for reverse transcription using SuperScript IV Reverse Transcription (Thermo Fisher Scientific). Quantitative PCR was performed using Thermal Cycler Dice TP850 (Takara Bio). The PCR primers are #12 for *mCherry* and #13 for human *GAPDH*. One of the 0 h cDNA samples was step-diluted by 0.5-fold and used as a standard for quantification. The relative value of *mCherry* divided by the relative value of *GAPDH* was averaged for the DMSO- and ASV-supplemented groups.

### Generating PD-1-mCherry-SMASh KI mice

For creating the homologous recombination vector, 1622 bp of murine genomic *Pdcd1* fragment, including the stop codon, was amplified using C57BL/6J genomic DNA as a template (KOD-plus-Neo, TOYOBO) and primers #14. The *Pdcd1* genomic DNA fragment was inserted into the HincII site of pUC118 using Mighty Cloning Reagent Set (Takara Bio). An EcoRV site was introduced upstream of the stop codon using the KOD-Plus Mutagenesis Kit and primers #15. The *mCherry*-*SMASh* fusion fragment was amplified by KOD-plus-Neo and primers #16. These fragments were inserted into the EcoRV site of pUC118-PD-1 using NEBuilder HiFi DNA Assembly Master Mix (NEB, MA) to produce the p*PD-1-mCherry-SMASh-*targeting vector. The DNA fragments, including the guide RNA sequence for *Pdcd1* (#17), were inserted into the *Bbs*I site of p*X330-U6-Chimeric_BB-CBh-hSpCas9* (Addgene #42230) to produce p*X330-PD-1*. The sequences of the targeting vector and gRNA-producing vector were confirmed by Sanger sequencing. B6J-S1 male embryonic stem cells (ESCs) (40) were cultured in ESC medium (Knockout DMEM, Thermo Fisher Scientific) supplemented with 15% Knockout Serum Replacement (Thermo Fisher Scientific), Glutamax (Thermo Fisher Scientific), MEM non-essential amino acids (Thermo Fisher Scientific), 1 mM sodium pyruvate (Thermo Fisher Scientific), penicillin-streptomycin, 10^4^ U/ml leukemia inhibitory factor, 300 μM CHIR99021 (GSK-3 inhibitor, Wako), and 100 μM PD0325901 (MEK inhibitor, Wako) on C3H-Neo embryonic fibroblasts treated with 10 μg/ml mitomycin C for 3 h before use. The p*PD-1-mCherry-SMASh* homologous recombination vector and the p*X330-PD-1* plasmids were electroporated into B6J-S1 ESCs simultaneously using Neon Transfection System at 1400 V, 10 ms, 3 times (Thermo Fisher Scientific, MA). Colonies from a single cell were picked up and the transformants were confirmed for 5′ and 3′ homologous recombination by PCR using primers #18 and #19 and genomic DNA as the template, respectively. For the transformants in which homologous recombination occurred, the percentage of cells with 40 chromosomes was examined, and the clones with >80% of cells with 40 chromosomes were used to generate chimeric mice. Chimera mice were produced as described in previously (39,41). The recombinant ESCs were treated with Accutase, and 5−8 cells were aggregated with a ICR 8-cell/morula-stage embryo after the removal of the zona pellucida using EmbryoMax Acidic Tyrode's solution (Thermo Fisher Scientific) and incubated at 37°C with 5% CO_2_ overnight. Next day, aggregated embryos with ESCs were transferred into pseudopregnant ICR (Clea Japan, Tokyo, Japan). The chimera mouse was bred with C57BL/6J WT females (Japan SLC Inc., Shizuoka, Japan) to obtain heterozygous mutant mice. Homozygous mutant mice were obtained by intercrossing heterozygous mutant mice.

### Culture of splenocytes

Splenocytes were harvested after euthanizing the mice. Splenocytes were isolated in RPMI 1640 medium, and the cells were collected through a 70 μm filter. Splenocyte culture was performed at a density of 2 × 10^6^ cells/ml in RPMI 1640 with 10% FBS, Glutamax and penicillin-streptomycin and incubated at 37°C in a 5% CO_2_ containing atmosphere. To activate T cells, 0.5 μg/ml concanavalin A was added to the medium for 3 days.

### Inoculation of MC-38 colon adenocarcinoma into mice and ASV/GRV or anti-PD-1 antibody treatment

MC38 cells were passaged one day before inoculation, and 1 × 10^6^ cells per head were inoculated subcutaneously in anesthetized female mice at the age of 4–6 months using syringes fitted with 23G needles (Terumo, Tokyo, Japan). The composition of the anesthetic was medetomidine (Domitor; Meiji Seika Pharma, Tokyo, Japan) at 0.3 mg/kg body weight, midazolam (Dormicum; Astellas, Tokyo, Japan) at 4 mg/kg body weight, and butorphanol (Betolfal; Meiji Seika Pharma) at 5 mg/kg body weight. Asunaprevir (ASV) (20 mg/kg/day) or grazoprevir (GRV) (10 mg/kg/day) administration by intraperitoneal injection was started 4–5 days after inoculation of MC-38 cells, which is the time point at which the tumor size reached more than 45 mm^3^. ASV and GRV were dissolved in DMSO to obtain 80 mg/ml and 40 mg/ml, respectively, and vortexed with nine times the volume of corn oil to form an emulsion; 50 ul of each solution was then administered intraperitoneally. The control group was administered DMSO adjusted in the same manner. Anti-PD-1 antibody (InVivoMAb anti-mouse PD-1, Bio X Cell; NH) (10 mg/kg/day) and isotype control (InVivoMAb polyclonal Armenian hamster IgG, Bio X Cell) (10 mg/kg/day) was injected intraperitoneally on day 3, 6 and 9 after MC-38 inoculation. Tumor sizes were measured everyday using a digital caliper. The tumor volume was calculated by applying the following formula: volume = (length of longer axis) × (length of shorter axis)^2^ × 0.52 (42,43). The endpoints of the cancer transplantation experiment were a tumor size of 2000 mm^3^, weight loss of 20% or more from the start of the experiment, and debilitation.

### Transplantation of BMCs into mice

BMCs from female mice were prepared as described previously (44). Briefly, BMCs were collected from the femurs and tibias of mice. After washing, the cells were resuspended in HBSS (1 × 10^7^ cells/ml). Recipient mice were irradiated with 9.5 Gy gamma rays at least 4 h before transplantation. It was confirmed that all mice died without transplantation when irradiated with this dose. Next, 1 × 10^6^ cells resuspended in 0.1 ml of BMC suspension were transplanted from an orbital venous plexus into anesthetized WT C57BL/6J female mice using a Myjector 27G syringe with a fixed needle (Terumo, Tokyo, Japan).

### Chimerism of the bone marrow transfer donor mice

The tail vein of the mice was nicked with a razor blade, and peripheral blood was collected in hematocrit tubes. Peripheral blood was collected into 1.5 mL plastic tubes from the hematocrit tubes and genomic DNA was purified using the Monarch Genomic DNA Purification Kit (NEB) as per manufacturer's instructions and the concentration of genomic DNA was measured using a Qubit fluorometer (Thermo Scientific). Twenty nanogram of genomic DNA was used for quantitative PCR for *mCherry* in KI cells and the amount of *Bcl2* was used as the reference using Thermal Cycler Dice TP850 (Takara Bio). The PCR primers are listed in #20 and #21 in Supplementary Table S2, respectively. The genomic DNA derived from the peripheral blood of WT and KI mice was adjusted so that the content of KI genome was 0%, 46%, 77%, 88% and 100%, and a regression line was generated. The percentage of KI cells was estimated by fitting the regression line to the results of quantitative PCR using genomic DNA derived from the peripheral blood of mice transplanted with KI BMCs.

### Measurement of autoantibody levels in the peripheral blood

Peripheral blood was collected into 1.5 ml plastic tubes without EDTA or heparin, incubated at room temperature for 2 h, and then centrifuged at 11 000 × *g* for 5 min to collect the serum. The serum was stored at −80°C until the start of the experiment. LEBIS mouse anti-dsDNA ELISA Kit (FUJIFILM Wako Shibayagi, Gunma, Japan), LEBIS mouse anti-ssDNA ELISA Kit (FUJIFILM Wako Shibayagi), and mouse anti-nuclear Antibody (ANA) ELISA Kit (MyBioSource, CA) were used for the measurement of autoantibody levels as described in the manufacturer's instructions. For anti-dsDNA ELISA and anti-ssDNA ELISA, each sample was diluted 100- and 200-fold with dilution buffer in the kit to confirm the linearity of the assay. For ANA ELISA, each sample was diluted 8-fold with PBS. A standard curve was generated from the absorbance of the standard solution by fitting a cubic curve using JMP Pro (14.0.0) (SAS, NC). Based on the obtained curve, the concentration of each sample was calculated.

### Histological analyses

Mice were anaesthetized with 4% isoflurane. After confirming that they were unconscious, all the blood was taken from the left atrium. The organs were harvested after euthanasia. The collected tissues were fixed in 4% neutral buffered formalin and processed for paraffin embedding. Briefly, the tissues were washed with tap water, dehydrated with 70%, 80% 95% and 100% ethanol, and soaked in xylene three times. The tissues were then immersed in paraffin three times and embedded in paraffin. Sections of three-micrometer thickness were sliced and pasted on glass slides, followed by hematoxylin-eosin, periodic Schiff PAS, and Elastica-Masson Goldner staining, and observed under the microscope. The criteria for scoring each glomerulus were as follows: 0, no symptom; 1, slight cell proliferation and mild cell infiltration; 2, endocapillary and mesangial proliferation forming a lobular structure; and 3, crescent formation with hyalinosis (as described by Miyazaki et al (45)). The glomerulonephritis index was calculated based on the score of 20 randomly selected glomeruli. The criteria for scoring each ankle joint of the left hindlimb for arthritis were as follows: 0, no symptom; 1, slight fibrocartilage proliferation; 2, marked fibrocartilage proliferation; and 3, ankylosis (46).

### Statistical analysis

All statistical analyses were performed using GraphPad Prism 9.1.0 (GraphPad Software, CA). The statistical method used, types of error bars, and sample size are annotated in each figure legend. A significant difference was considered when the *P*-value was <0.05.

## RESULTS

### Localization and dynamics of PD-1 fused to mCherry and SMASh tag in T-lymphoma cell lines

**Figure 1.**
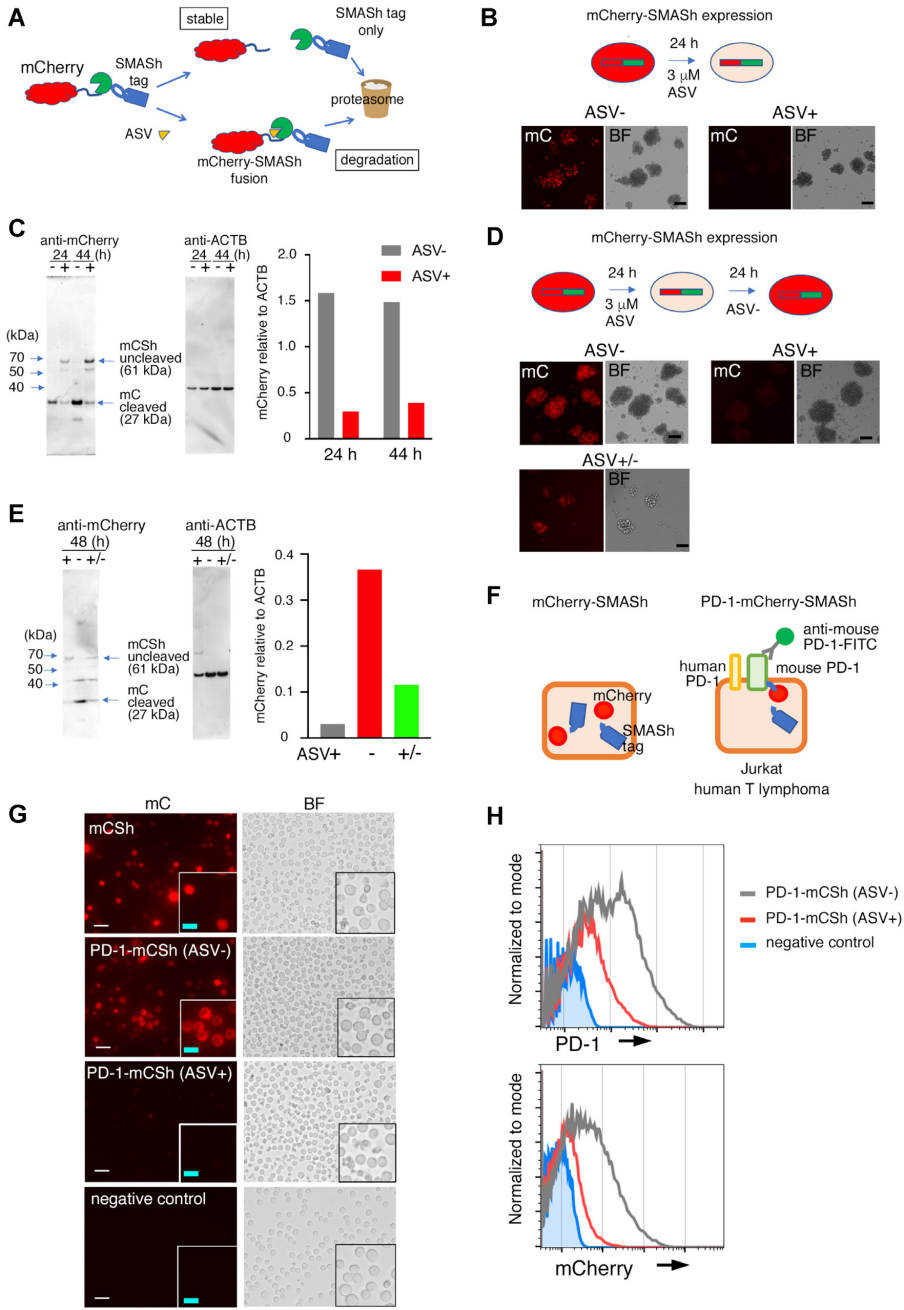
Dynamics of PD-1-mCherry-SMASh fusion protein in EL4 murine T-lymphoma cells and Jurkat human T-lymphoma cells treated with asunaprevir (ASV). (**A**) Schematic diagram of the SMASh degron system on mCherry fusion protein. (**B**) Fluorescent mCherry (mC) and bright-field (BF) images of mCherry-SMASh-expressing EL4 cells with (+) or without (–) ASV treatment for 24 h. Scale bars: 10 μm. (**C**) Western blotting of cell lysate proteins from the *mCherry-SMASh* vector-transfected EL4 cells using anti-mCherry and anti-β-actin antibodies. The mCherry levels relative to β-actin (ACTB) were quantified as shown in the graph. The 24- and 44-hour samples comprised the polyclonal cells that were treated at the same time; the experiment was performed once. (**D**) Fluorescent mCherry and BF images in mCherry-SMASh-expressing EL4 cells. (Top left) without ASV (–), (top right) 48 h after addition of ASV (+), (bottom) cultured for 24 h with ASV, and then ASV was removed and cultured for 24 h (+/–). Scale bars: 10 μm. (**E**) Western blotting of cell lysate proteins from the same cells in (D) using anti-mCherry and anti-β-actin (ACTB) antibodies. The level of mCherry relative to ACTB was quantified and shown in the graph. ASV+, ASV-, and ASV+/- samples comprised the polyclonal cells that were treated at the same time; the experiment was performed once. (**F**) A schematic diagram of mCherry-SMASh (left) and PD-1-mCherry-SMASh (right) fusion protein in a cell without ASV. (**G**) Fluorescent mCherry (mC) and bright-field (BF) images of Jurkat cells expressing mCherry-SMASh (mCSh), PD-1-mCherry-SMASh (PD-1-mCSh) without ASV (ASV–), PD-1-mCherry-SMASh (PD-1-mCSh) with ASV (ASV+) and the backbone vector (negative control) 1 day after transfection. The bottom right insert of each image corresponds to a magnified view. White scale bars: 20 μm, light blue scale bars: 10 μm. (**H**) Flow cytometric analyses of cell-surface PD-1 (top) and mCherry (bottom) in PD-1-mCherry-SMASh-expressing Jurkat cells without ASV (ASV–, grey) or with ASV (ASV+, red) for 3 days and backbone vector (negative control, blue).

To confirm the function of the SMASh degron system in a murine T-cell line (Figure 1A and Supplementary Figure S1), EL4 cells derived from murine T-cell lymphoma were transfected with the mCherry-SMASh degron tag expression vector, which was integrated into the ROSA26 locus (Supplementary Figures S2A–C). Twenty-four and forty-four hours after the addition of ASV, the expression level of mCherry in the cells was reduced to 19% and 26%, respectively, compared to that in control cells (Figure 1B and C). Following ASV treatment, the levels of cleaved mCherry (27 kDa) reduced and those of the uncleaved mCherry-SMASh fusion protein (61 kDa) increased compared to those in untreated cells (Figure 1C), indicating that ASV treatment leads to the degradation of the fusion protein. Next, we examined whether mCherry levels increased after the removal of ASV. The expression of mCherry 24 h after the removal of ASV (+/– in Figure 1D and E) increased 3.7-fold compared to that in the presence of ASV (+ in Figure 1D and E). The time course of mCherry degradation with ASV treatment and re-expression with the removal of ASV was also examined by flow cytometry (Supplementary Figure S3). The mean fluorescence intensity of mCherry continued to decrease until 96 h after adding ASV to 24.6 ± 1.1% compared to that treated with the vehicle (Supplementary Figure S3A). The fluorescence intensity of mCherry was recovered 120 h after removal of ASV following 48 h of exposure to ASV to 83.3 ± 0.7% (Supplementary Figure S3B). During the reduction of the fluorescence intensity of mCherry with ASV, the amount of mRNA of mCherry in EL4 treated with ASV was comparable to that treated with the vehicle (Supplementary Figure S3C).

Furthermore, we examined whether the mouse PD-1-mCherry fusion protein expressed using the transient Pdcd1-mCherry-SMASh expression vector (Supplementary Figure S2D) localized to the cell surface in Jurkat human T-lymphoma cells (Figure 1F). In cells transfected with the mCherry-SMASh expression vector, mCherry was detected throughout the entire cell (Figure 1G). In cells harboring the Pdcd1-mCherry-SMASh expression vector, mCherry was localized outside the nucleus (Figure 1G). We examined whether mouse PD-1-mCherry-SMASh levels decreased upon addition of ASV, similar to mCherry-SMASh (Supplementary Figure S2E). In Jurkat cells cultured in an ASV-containing medium for 1 day, mCherry levels were reduced compared to those in cells cultured in the absence of ASV (Figure 1G). Immunostaining revealed that mouse PD-1 was detectable on the cell surface, and its signal intensity had a positive correlation with mCherry signal intensity (Supplementary Figure S4). Therefore, we confirmed that mouse PD-1-mCherry localized to the cell surface, and the N-terminus of PD-1 was exposed to the extracellular space in the absence of ASV. Three days after the addition of ASV, the average signal intensities of PD-1 and mCherry in ASV-treated cells were 28% and 15% (PD-1), and 30% and 7.4% (mCherry), respectively, of those in cells cultured in the absence of ASV in the two experiments (Figure 1H, the histograms of the former results were shown). This result indicated that mouse PD-1-mCherry-SMASh decreased with the addition of ASV, similar to mCherry-SMASh.

### PD-1-mCherry-SMASh was attenuated in homozygous knock-in (KI) splenocytes by the addition of ASV *in vitro*

**Figure 2.**
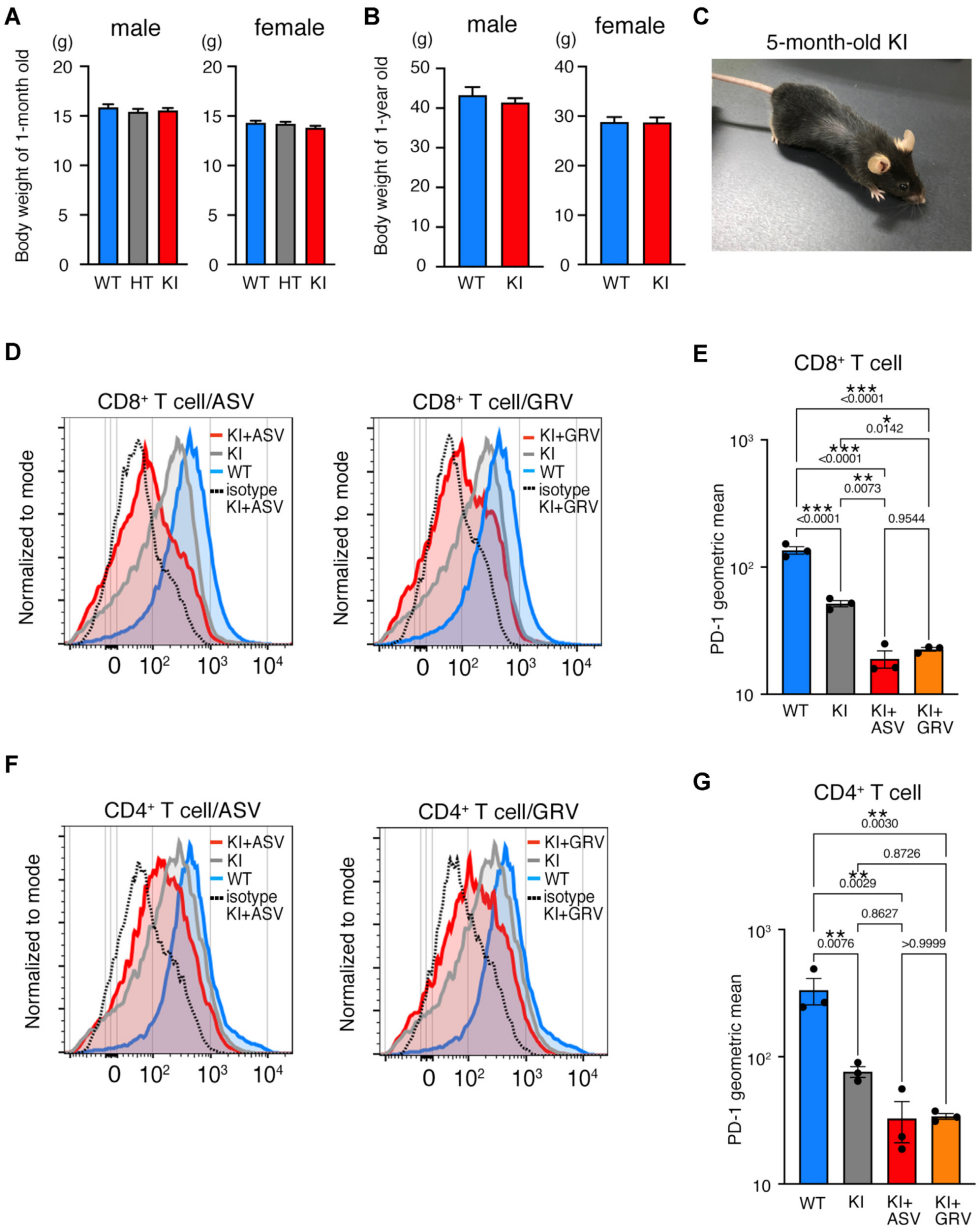
PD-1 and mCherry in PD-1-mCherry-SMASh homozygous mutant (KI) splenocytes in the presence of asunaprevir (ASV) or grazoprevir (GRV). (A, B) Body weights of wild-type (WT), heterozygous mutant (HT), and homozygous mutant (KI) mice at ages of 1 month (**A**) and 1 year (**B**). The numbers of individuals are as follows: 1-month-old males: 90 WT, 104 HT and 145 KI; 1-month-old females: 91 WT, 106 HT, and 121 KI; 1-year-old males: 6 WT and 29 KI; 1-year-old females: 10 WT and 14 KI. Data are represented as the mean + standard deviation (SD). (**C**) Normal appearance of 5-month-old KI mouse. (**D**) Flow cytometric analyses of PD-1 expression in WT, KI without ASV (KI), and KI with ASV (KI+ASV) CD8^+^ T cells stimulated concanavalin A (Con A). The curves of isotype control (dotted line) are the result of the same sample as KI+ASV/GRV in the graph. The experiment was performed independently for splenocytes from three mice per each genotype twice. The data show the results of one experiment. (**E**) The geometric means of fluorescence intensity of PD-1-FITC in CD8^+^ T cells of each genotype shown in (D). The results of the statistical test were comparable for the other experiment. (**F**) Flow cytometric analyses of PD-1 expression in WT, KI without ASV (KI), and KI with ASV (KI+ASV) CD4^+^ T cells stimulated Con A. The experiment was performed independently for splenocytes from three mice per each genotype twice. The data show the results of one experiment. (**G**) The geometric means of fluorescence intensity of PD-1-FITC in CD4^+^ T cells of each genotype shown in (F). The statistical test showed that the results of the other experiment were comparable. In (E) and (G), a geometric mean of each sample was calculated by subtraction of the geometric mean stained with isotype control from that stained with anti-PD-1 antibody. Data are represented as the mean + standard error (SE). *P* values obtained by one-way ANOVA were shown in the graph. *P < 0.05; **P < 0.01; ***P < 0.001.

We generated homozygous KI mice harboring mCherry-SMASh (mCSh). mCherry-SMASh was integrated downstream of *Pdcd1* by homologous recombination in C57BL/6 embryonic stem (ES) cells (Supplementary Figure S5A). The genotypes of the offspring produced by intercrossing heterozygous mutant (HT) mice followed the Mendelian ratio (Supplementary Figure S5B and Table S1). The body weights of 1-year-old and 1-month-old KI mice were comparable to those of WT and HT mice (Figure 2A and B). KI mice were fertile, and no apparent disease was observed at 5 months (Figure 2C).

Splenocytes from WT and KI mice were stimulated with concanavalin A in vitro for 3 days to evaluate the effect of ASV on PD-1 expression in CD8^+^ and CD4^+^ T cells (Figure 2D–G and Supplementary Figure S6). We also used GRV to induce the SMASh degron system (Figure 2D-G). GRV also inhibits the NS3/4A protease and has a lower Ki value than ASV, with Ki values of 0.01 nM for HCV genotypes of 1a and 1b (47), whereas ASV exhibits Ki values of 0.7 and 0.3 nM against 1a and 1b, respectively (48). The signal intensity of PD-1 in ASV-treated KI (KI+ASV) and GRV-treated KI (KI+GRV) CD8^+^ T cells was significantly reduced compared to that in WT cells (Figure 2D and E). Contrary to expectations, the amount of PD-1 in KI CD8^+^ T cells was reduced compared to that in WT, even without the addition of ASV or GRV. The signal intensity of PD-1 in KI+ASV and KI+GRV CD4^+^ T cells was reduced compared to that in WT cells (Figure 2F and G). However, treatment with ASV or GRV did not significantly decrease PD-1 expression in KI CD4^+^ T cells without treatment.

The effects of ASV and GRV on the expression of PD-1 in WT T cells were also examined. In WT CD8^+^ and CD4^+^ T cells, there was no difference in the expression of PD-1 between ASV/GRV-treated and untreated cells (Supplementary Figure S7).

### Growth of MC-38 colon adenocarcinoma cells introduced into KI mice was repressed with ASV or GRV treatment

**Figure 3.**
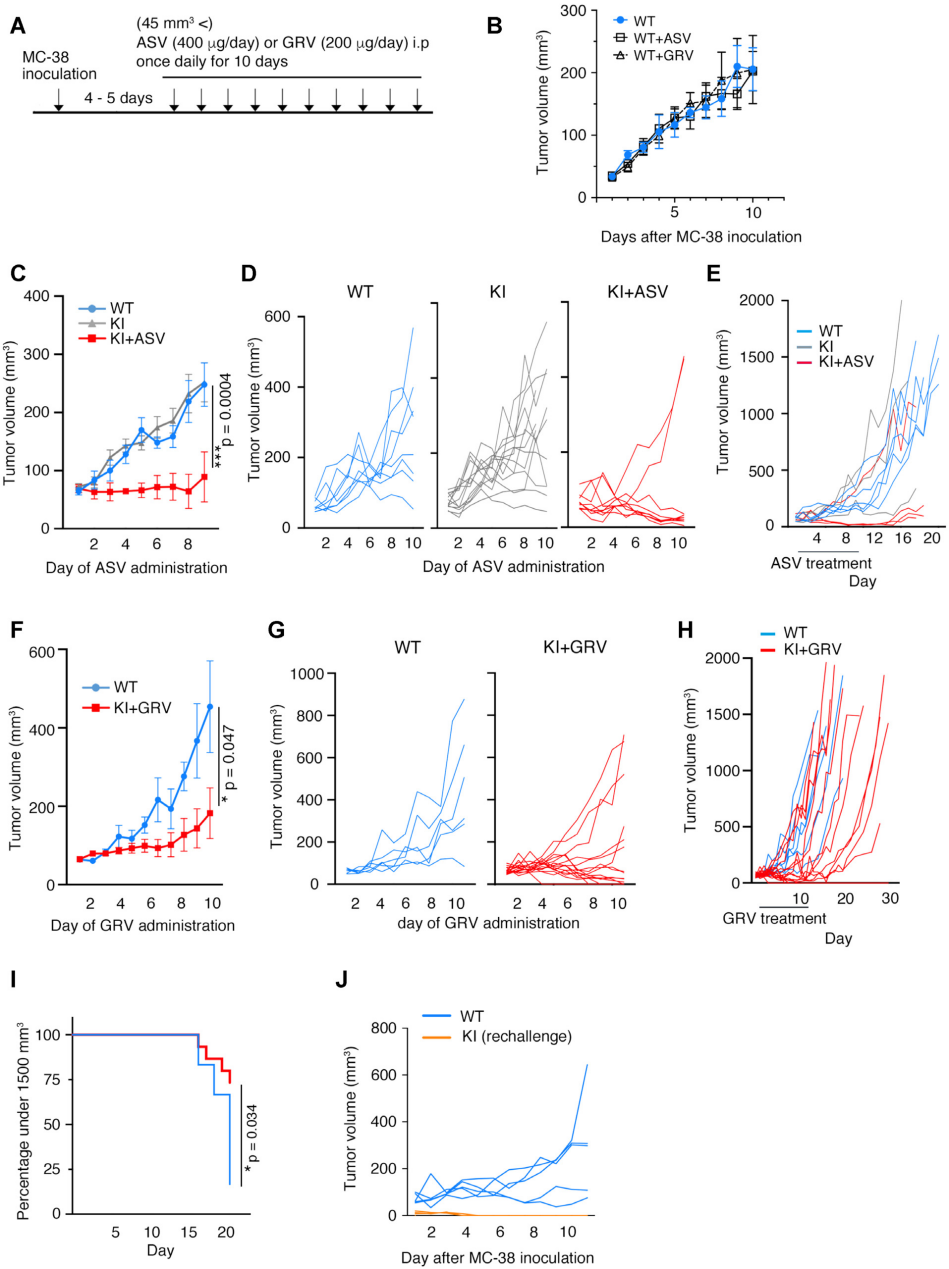
Growth of MC-38 cells was repressed in PD-1-mCherry-SMASh knock-in (KI) mice treated with asunaprevir (ASV) or grazoprevir (GRV). (**A**) Schedule of the experiments. (**B**) Growth curve of MC-38 cells in wild-type mice treated with ASV (WT+ASV, *n* = 6), with GRV (WT+GRV, n = 6) or without treatment (WT, *n* = 6). One million MC-38 cells were injected subcutaneously, and MC-38 cell masses were measured from day 4. When cell masses reached a volume greater than 45 mm^3^, the administration of ASV, GRV or was initiated. The experiment was performed once. (**C**) Growth curve of MC-38 cell masses in WT (light blue, *n* = 8), KI (grey, *n* = 13), and KI+ASV mice (red, *n* = 9). Data are represented as mean ± standard error (SE). Transplantation of MC-38 cells was performed in two separate experiments, and the results are represented as a single graph. (**D**) Individual growth curve of MC-38 cell masses in the same mice shown in (C). (**E**) Long-term observation of MC-38 proliferation in each experimental group (WT, *n* = 5; KI, *n* = 3; KI + ASV, *n* = 4). The experiment was performed once. (**F**) Growth curve of MC-38 cell masses in WT (light blue, *n* = 6) and KI mice treated with GRV (KI + GRV) (red, *n* = 15). Data are represented as mean ± SE. MC-38 cells were transplanted in two separate experiments, and the results are presented in a single graph. (**G**) Individual growth curve of MC-38 cell masses in the same mice are shown in (F). (**H**) Long-term observation of MC-38 proliferation in each experimental group. (**I**) Time course of MC-38 cell mass until the volume reached 1500 mm^3^. (**J**) Cell growth curve of MC-38 cells reintroduced into KI+GRV mice after they were previously eliminated. No GRV treatment was given at rechallenge (WT, *n* = 5; KI rechallenge *n* = 4). The experiment was performed once. ***P < 0.001; *P < 0.05; two-way ANOVA with the Geisser-Greenhouse correction (C, F) and Gehan–Breslow–Wilcoxon test (I). Degrees of freedom and *F* values are shown in Supplementary Table S5.

MC-38 cells (1 × 10^6^ cells) were subcutaneously injected into KI mice to evaluate the effect of ASV and GRV on tumor growth. The experimental schedule is shown in Figure 3A. ASV administration (20 mg/kg/day) was started 4–5 days after injection of MC-38 cells, which is the time point when the tumor size reached more than 45 mm^3^. The proliferation of MC-38 cells in WT mice upon ASV administration was comparable to that of WT mice without ASV treatment (Figure 3B), suggesting that ASV does not exhibit any inhibitory effect on MC-38 proliferation. Growth of MC-38 cells in KI mice upon ASV treatment (KI+ASV) was repressed compared with that in WT mice and KI mice treated with the vehicle (KI) (Figure 3C and D). After the termination of ASV administration, growth of MC-38 cells was observed even in KI+ASV mice that had not eliminated MC-38 cells; however, the growth rate was significantly repressed compared to that in KI (Figure 3E). Upon injection of 3 × 10^5^ MC-38 cells, the cancerous cells disappeared in 56% of the KI+ASV mice (Table 1), and mice were remained tumor-free for at least two months. The effect of ASV on the reduction of MC-38 cell growth in KI mice was comparable to that in WT mice with anti-PD-1 antibody treatment (Supplementary Figure S8).

**Table 1.** The number and percentage of mice that survived more than 2 months after MC-38 elimination

Drug	MC-38	WT	KI	KI + drug
ASV (20 mg/kg/day)	3 × 10^5^	1/12 (8%)	4/13 (31%)	5/9 (56%)
ASV (20 mg/kg/day)	1 × 10^6^	0/12 (0%)	3/17 (18%)	3/11 (27%)
GRV (10 mg/kg/day)	1 × 10^6^	0/14 (0%)	ND	4/15 (27%)

Administration of GRV (10 mg/kg/day; KI + GRV) was also effective in inhibiting MC-38 proliferation (Figure 3F–I). The proliferation of MC-38 cells in WT mice upon GRV administration was comparable to that of WT mice with ASV treatment (Figure 3B). When 1 × 10^6^ MC-38 cells were injected into KI + GRV mice, 4 out of 15 mice eventually eliminated them (Table 1). These 4 KI + GRV mice were injected with 1 × 10^6^ MC-38 cells again, two months after the initial elimination of MC-38 cells. The MC-38 cells were eliminated again without GRV administration (Figure 3J), suggesting that cancer immunity was established.

### Analyses of immune cells in the tumor microenvironment of KI mice upon ASV treatment

**Figure 4.**
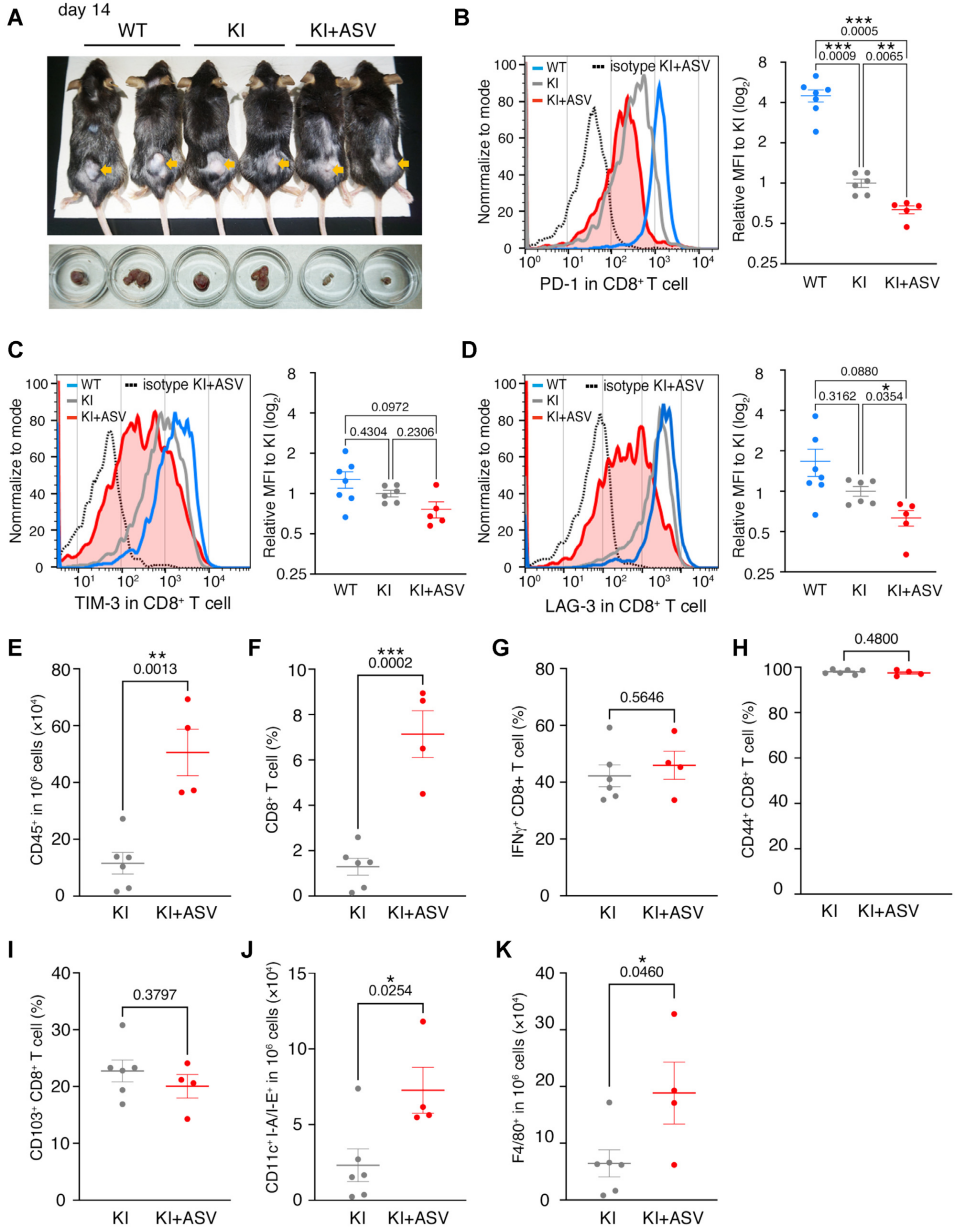
Tumor-infiltrating immune cells in KI mice with asunaprevir (ASV) treatment. (**A**) Examples of mice and cell masses analyzed by FACS at day 14 after initiation of ASV treatment. (B–D) Cell -surface expression of PD-1 (**B**), TIM-3 (**C**) and LAG-3 (**D**) in CD8^+^ T cells in the microenvironment of MC-38 cell masses. The curves for isotype control (dotted line) are the results for the same sample as KI+ASV presented in the graph. WT (blue, *n* = 7), KI (grey, *n* = 6), KI+ASV (red, *n* = 5), biologically independent samples. The mean for each sample was calculated by subtracting the mean for sample stained with isotype control from that for the sample stained with anti-PD-1 antibody. Transplantation of MC-38 cells was performed in two separate experiments, and the results are represented as a single graph. (**E**) The numbers of tumor-infiltrating CD45^+^ immune cells analyzed by flow cytometry. KI (grey, *n* = 6) and KI+ASV (red, *n* = 4), biologically independent samples. (F–I) The ratios of tumor-infiltrating CD8^+^ T cells in CD45+ cells (**F**), IFNγ^+^CD8^+^ effector T cells (**G**), CD44^+^CD8^+^ effector T cells (**H**) and CD103^+^CD8^+^ residual memory T cells (**I**) analyzed by flow cytometry. KI (grey, *n* = 6), KI+ASV (red, *n* = 4), biologically independent samples. (J, K) The numbers of tumor infiltrating dendritic cells (**J**) and macrophages (**K**) in 10^6^ cells of microenvironment. KI (grey, *n* = 6), KI+ASV (red, *n* = 4), biologically independent samples. Data are represented as mean ± standard error (SE). The experiment was conducted twice independently, and the results are presented in a single graph. *P* values obtained by Brown-Forsythe Welch ANOVA tests (B–D) and unpaired two-tailed *t*-tests (E–K) were shown in the graph. *P < 0.05; **P < 0.01; ***P < 0.001.

Immune cells in the tumor microenvironment of the MC-38 tumors were analyzed on day 14 (Figure 4A and Supplementary Figure S9A-S9C). PD-1 expression levels were reduced in KI+ASV CD8^+^ T cells compared to that in KI CD8^+^ T cells and to 10% compared to that in WT CD8^+^ T cells (Figure 4B). The expression of other exhausted markers on the CD8^+^ T-cell surface besides PD-1, TIM-3 and LAG-3 was analyzed (Figure 4C and D), and the intensity of TIM-3 signals in KI+ASV CD8^+^ T cells was comparable to that in KI and WT cells (Figure 4C). The intensity of LAG-3 was decreased in KI+ASV CD8^+^ T cells compared to that in KI cells (Figure 4D).

The count of immune cells associated with the cancerous immune system was examined in the tumor microenvironment of KI and KI+ASV mice. CD45^+^ cells and CD8^+^ T cells in the KI+ASV microenvironment markedly increased compared to those in the KI microenvironment (Figure 4E and F), suggesting that the infiltrating activity of immune cells and CD8^+^ T cells was increased in KI+ASV mice. The populations of IFNγ^+^ (effector T) cells, CD44^+^ (effector T) cells, and CD103^+^ (residual memory T) cells among CD8^+^ T cells in KI+ASV mice were comparable to those in KI mice (Figure 4G–I). The counts of CD11c^+^ I-A/I-E^+^ dendritic cells and F4/80^+^ macrophages in the KI+ASV microenvironment increased compared to those in the KI microenvironment (Figure 4J and K). These data suggested that the infiltration of cells related to cancer immunity was enhanced in the KI + ASV microenvironment compared to that in the KI microenvironment, whereas the populations of effector and memory T cells were the same between KI + ASV and KI microenvironments. We also examined the immune cells in WT and WT + ASV microenvironments and did not observe any differences (Supplementary Figures S10A–H).

### Transplantation of KI bone marrow cells (BMCs) into WT mice led to MC-38 cell rejection upon GRV administration

To confirm the effect of the immune cells on MC-38 cell rejection, KI BMCs or WT control BMCs were transplanted into WT mice irradiated with 9.5 Gy of gamma rays. Two irradiated mice that were not transplanted with BMCs died by day 10. Thirty-five days after transplantation, the chimerism of the peripheral blood cells in transplanted mice was analyzed using quantitative PCR (49). The percentage of KI BMC-derived DNA was calculated to be >100% on average (Supplementary Figure S11), indicating that almost all peripheral blood cells were replaced by KI cells.

**Figure 5.**
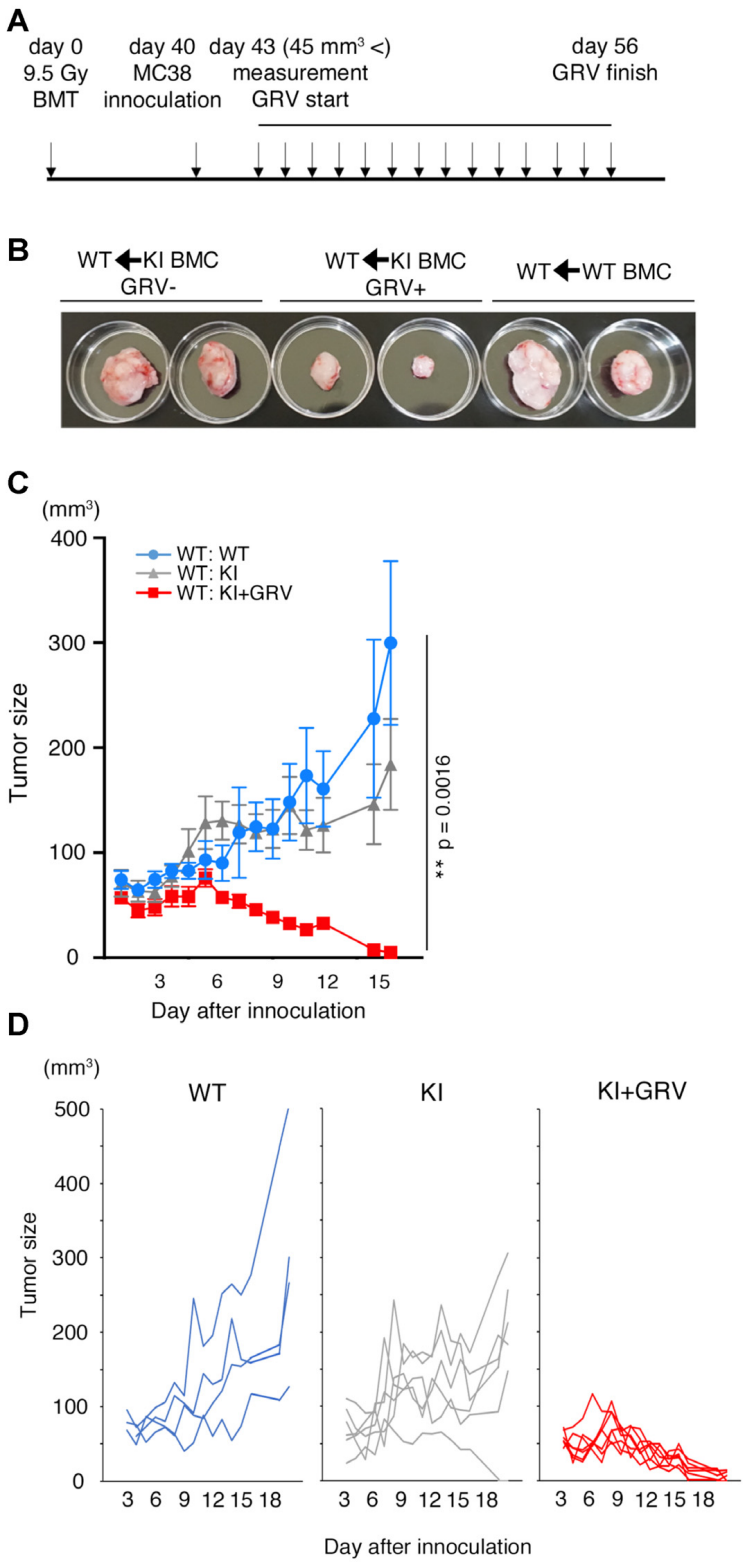
Growth of MC-38 cells was repressed in wild-type (WT) mice upon grazoprevir (GRV) administration after transplantation of PD-1-mCherry-SMASh knock-in (KI) bone marrow cells (BMCs). (**A**) Schedule for the experiments. Donor BMCs were transplanted through an orbital venous plexus into recipient WT mice irradiated with a lethal dose of radiation. Forty days after BMC transplantation, MC-38 cells were injected into the mice, and GRV was administered to them. (**B**) MC-38 cell masses in WT mice transplanted with WT BMCs (right), WT mice transplanted with KI BMCs and treated with the vehicle (GRV–, left), and WT mice transplanted with KI BMCs and treated with GRV (GRV+, center), 28 days after initiation of GRV treatment. (**C**) Growth curve of MC-38 cell masses in WT mice transplanted with WT BMCs (WT: WT, blue, *n* = 4), WT mice given vehicle (DMSO) after transplantation of KI BMCs (WT: KI, grey, *n* = 6) and WT mice given GRV after transplantation of KI BMCs (WT: KI + GRV, red, *n* = 7). Data are represented as mean ± standard error (SE). **P < 0.01; two-way ANOVA with the Geisser–Greenhouse correction. Degrees of freedom and *F* values are shown in Supplementary Table S5. The experiment was performed twice. The data show the results of one experiment; the statistical test showed that the results of the other experiment were comparable. (**D**) Individual growth curve of MC-38 cell masses in the same mice shown in (C).

MC-38 cells (1 × 10^6^) were subcutaneously injected into the blood chimera mice 40 days after BMC transplantation. When the tumor size grew to a volume larger than 45 mm^3^, treatment with GRV was initiated for those mice (Figure 5A). The proliferation of MC-38 cells was inhibited by GRV in WT mice transplanted with KI BMCs (WT: KI + GRV) compared to that in untreated WT mice transplanted with WT BMCs (WT:WT) or untreated WT mice transplanted with KI BMCs (WT: KI) (Figure 5B–D). In addition, MC-38 cells were eliminated in three out of seven mice transplanted with KI BMCs, and MC-38 cells did not regrow until 6 months after GRV administration was terminated.

### Normal levels of autoantibodies and minor symptoms of immune-related adverse events in KI and KI+ASV mice.

**Figure 6.**
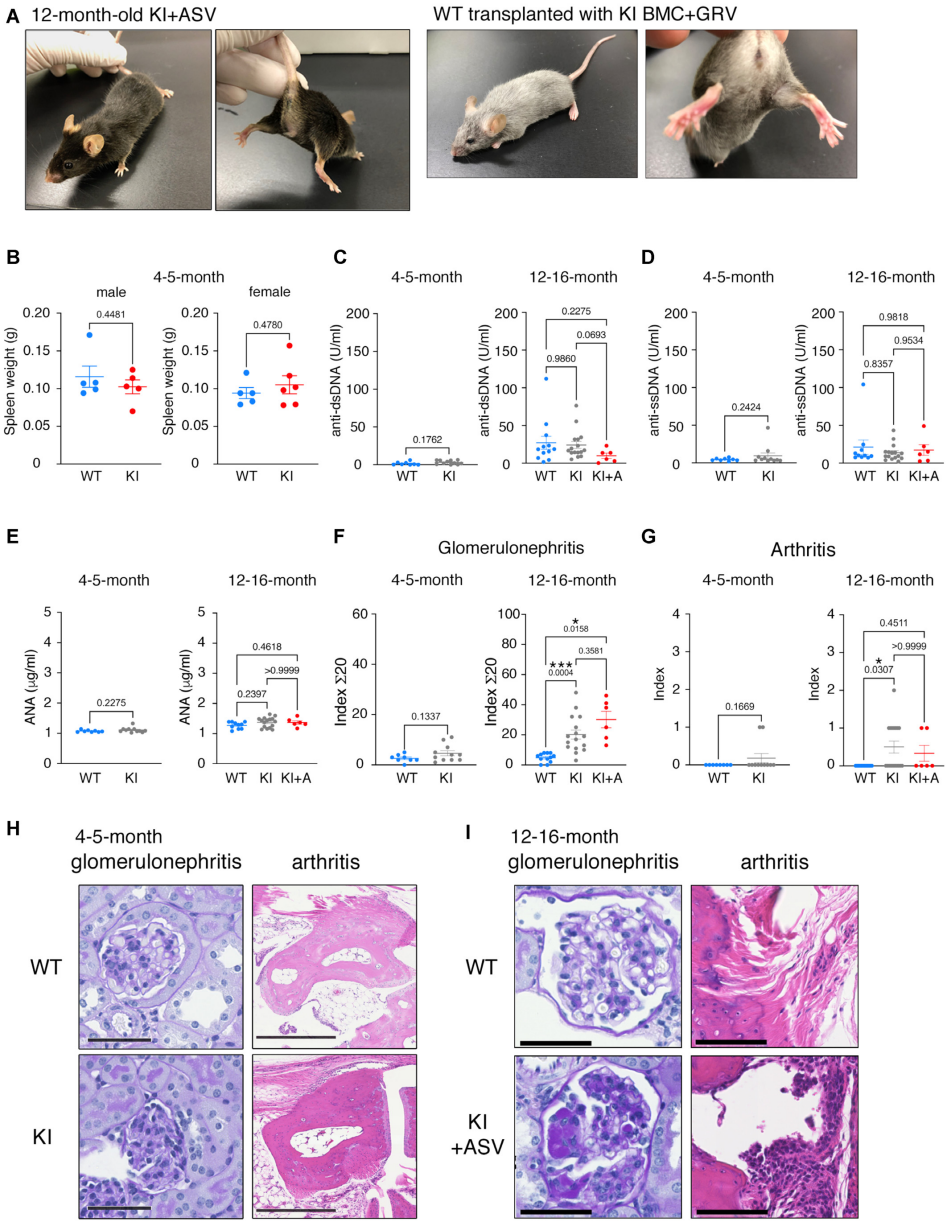
Analyses of autoimmune disease-like symptoms in KI mice with or without injection of asunaprevir (ASV) or grazoprevir (GRV). (**A**) Appearance of 12-month-old KI mouse treated with ASV at the age of 4 months. (**B**) Spleen weight of 4–5-month-old male and female WT (light blue, *n* = 5) and KI (red; males, *n* = 5; females, *n* = 6) mice. (C–E) Autoantibody titers in the sera of WT, KI and KI + ASV mice measured using ELISA. Anti-double strand (ds) DNA antibody (**C**), anti-single strand (ss) DNA antibody (**D**) and anti-nucleus antibody (ANA) (**E**) were quantified. Four- to five-month-old mice: WT, *n* = 8; KI, *n* = 11. Twelve- to 16-month-old mice: WT, *n* = 12 (C), n = 10 (D, E); KI, *n* = 16; KI + ASV, *n* = 6. (**F**, **G**) Scores of histological analyses of autoimmune diseases in 4–5-month- and 12–16-month-old WT (blue), KI (grey), or KI + ASV (red) mice. Four- to five-month-old mice: WT, *n* = 8; KI, *n* = 11. Twelve- to sixteen-month-old mice: WT, *n* = 12; KI, *n* = 16; KI + ASV, *n* = 6. Data are represented as mean ± SE. (**H**, **I**) Photomicrographs of histopathological manifestations in 4–5-month-old (H) and 12–16-month-old (i) mice analyzed in (E). Pictures show the most severe parts of each stage. Scale bars: 50 μm for glomerulonephritis, 500 μm for arthritis. *P* values obtained by unpaired two-tailed *t*-tests (4–5-month) and Brown-Forsythe Welch ANOVA tests (12–16-month) were shown in the graph. *P < 0.05; ***P < 0.001.

PD-1 homozygous knockout (KO) mice develop autoimmune diseases after 6 months of age (50–52). The development of arthritis and glomerulonephritis is prominent in PD-1 knockout mice. Therefore, we investigated the autoantibody titers and the development of these diseases in WT and KI mice. There was no observable abnormality in KI mice at the age of 4−5 months (Figure 2C). We also observed one-year-old KI mice treated with ASV at the age of 4–5 months with the same protocol shown in Figure 3 and WT mice at 4 months after transplantation of KI BMCs and treatment with GRV. There was no observable abnormality (Figure 6A), and no difference in WT and KI spleen weight at 4−5 months of age (Figure 6B) was observed. Serum anti-double strand (ds) DNA antibody, anti-single strand (ss) DNA antibody, and anti-nucleus antibody (ANA) concentrations were examined in WT and KI mice at 4–5 months and 12–16 months of age (Figure 6C–E), wherein no significant differences were observed.

We also conducted pathological analyses in these mice, and no significant difference in histopathological scores was observed between WT and KI mice at 4−5 months of age (Figure 6F-H). KI + ASV mice that received drug treatment were analyzed after 8 months to allow sufficient time for the onset of immune-related adverse events. The histopathological scores of KI + ASV and KI mice were higher than those of WT mice at the same age, indicating that KI + ASV and KI mice developed glomerulonephritis and arthritis at 12–16 months of age (Figure 6F, G and I). There was no significant difference between KI + ASV and KI mice, suggesting that the symptoms were due to leakage of the degron system rather than treatment (Figure 6F and G).

### The linker sequences between PD-1-mCherry and SMASh tag influenced protein degradation *in vitro*

**Figure 7.**
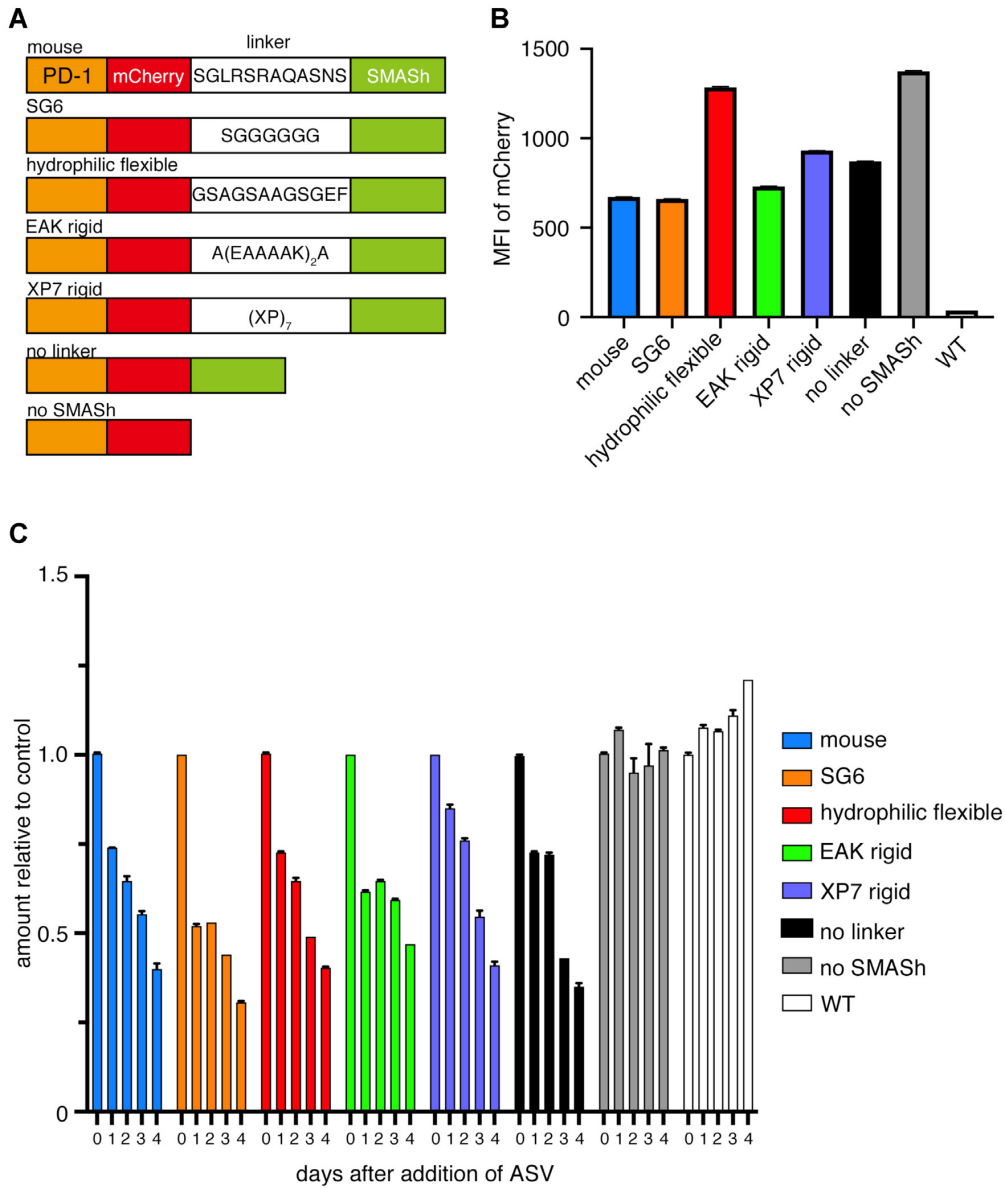
Difference of the amount of PD-1-mCherry protein using various linker between mCherry and SMASh. (**A**) Amino acid sequences of various linkers between PD-1 and SMASh tag. (**B**) Expression amount of mCherry without ASV treatment quantified by flow cytometry. (**C**) Time course of mCherry intensities after adding 10 μM ASV. The relative values to the fluorescence intensity of the samples to which DMSO was added are shown. The experiment was performed twice using three independent samples of polyclonal-derived cells. The data show the results of one experiment, and the result of the other experiment was comparable. The error bars are so short that the error bars are not visible in some samples (B, C).

The reason for the decrease in PD-1 expression even without ASV or GRV could be that the cleavage of the target sequence between PD-1-mCherry complex and NS3 protease in SMASh tag was not sufficient. This might be due to the structural problems between the target protein and NS3 protease. Therefore, we examined the expression level of PD-1-mCherry by stably expressing a construct with a hydrophilic or hydrophobic linker between the PD-1-mCherry complex and the SMASh tag in Jurkat cells (Figure 7A). The results showed that when the hydrophilic linker was inserted, the expression of PD-1-mCherry was equivalent to that without SMASh tag (Figure 7B), however, the degradation rate was about 40% with any linker (Figure 7C).

## DISCUSSION

In the present study, we employed the SMASh degron system to knockdown an endogenous target protein, PD-1, in mice. We were able to eliminate injected cancer cells using this experimental system in cultured cells and a mouse model. The growth of MC-38 cells injected in PD-1-mCSh KI mice was repressed by drug treatment. MC-38 cells completely disappeared in some KI mice treated with the drugs. The growth of MC-38 cells was also suppressed in bone marrow chimeric mice, which were generated by transplanting KI BMCs into WT mice irradiated with a lethal dose of radiation and treated with GRV. Therefore, the inhibition of MC-38 cell growth in KI mice upon drug treatment may be attributed to KI hemocytes. In the SMASh tag-based degron system, drug-meditated protein degradation is limited to the protein translated after drug administration. Nevertheless, as the half-life of PD-1 in activated cytotoxic T cells is approximately 3 h (53)—which is sufficiently shorter than the period of tumor treatment—this degron system may be effective in tumor treatment. In KI + ASV microenvironments, the numbers of DCs and macrophages were increased, while the ratio of IFNγ positive cells in CD8+ T cells was comparable to that in KI cells. The increase in IFNγ-positive cells following administration of anti-PD-1 antibody peaks on the second day after administration, after which the number of IFNγ-positive cells declines (54). In our experiment, MC-38 cells had already shrunk by the time flow cytometry was performed, and IFNγ levels may have peaked out.

PD-1 levels were reduced in KI mice without drug treatment (compared to that in WT mice), suggesting that the SMASh system was leaky. This might be due to incomplete cleavage of the degron tag from PD-1-mCherry. In several proteolysis-targeting chimera systems, the efficiency of protein knockdown has been improved by altering the structure of the linker between the target protein-binding molecule and the E3 ligase ligand (55). In our study, the decrease in mCherry in the absence of the drug could also be rescued by modifying the linker sequence.

The PD-1 level in T cells infiltrating the microenvironment of MC-38 cells injected into KI+ASV mice was approximately 50% of that in tumor-infiltrating T cells in mice without treatment. The results suggested that the reduced PD-1 levels in KI + ASV mice may be sufficient to inhibit MC-38 cell proliferation. As fluorescent proteins are not degraded efficiently by the proteasome in some conditions (56), PD-1-mCherry might not be degraded completely. In the next attempt, we aim to try to tag PD-1 directly with the SMASh tag to create a more effective PD-1 knockdown system. Alternatively, modification of the NS4 sequence, a degron in the SMASh tag, may also be necessary. In addition, in vitro study indicated that PD-1 on CD4+ T cells was not significantly decreased with ASV and GRV treatment. This may be due to differences in the amount of ASV and GRV taken up, the rate at which ASV and GRV are metabolized intracellularly, and the speed of protein degradation among cell types. The SMASh degron system may need to be optimized for different cell types.

As PD-1 transmits an inhibitory signal for the immune response, PD-1 knockout mice develop autoimmune diseases, such as lupus-like glomerulonephritis and arthritis, in the C57BL/6 background (51). PD-1 knockout mice also exhibit mild but consistent splenomegaly at 6 months (50). Despite the leaky nature of the degron system and reduction in PD-1 levels, in the present study, 4−5-month-old KI mice appeared healthy, with no evidence of splenomegaly and autoimmune diseases. At 1 year, however, both KI and KI + ASV mice developed glomerulonephritis and arthritis, similar to a lupus-like disease manifested by PD-1 knockout mice (51). As there was no significant difference between KI and KI + ASV mice, the symptoms of the disease were not due to the treatment, but due to leakage of the degron system. The aging-related symptoms may also be due to the leaky degron system; consequently, reducing the leakiness using the methods described above may be important for the long-term use of the degron system. The possibility that autoimmune disease was caused by the effects of ASV or GRV cannot be ruled out based on this study alone. However, autoimmune diseases have not been reported as a side effect of ASV for HCV treatment in humans. Therefore, ASV or GRV alone is probably not likely to cause autoimmune diseases.

This system could be exploited for a better understanding of PD-1 function for improved immunotherapy. Although the mechanism by which PD-1 affects CD8^+^ T-cell fate determination, memory cell function, or effector CD8^+^ T-cell reactivation remains to be addressed (57), it could be analyzed in such an experimental system via PD-1 expression regulation in time. For example, although PD-1 is expressed on the surface of T cells in the anergy and exhausted states, it remains unclear whether PD-1 is involved in the maintenance of the states or whether anti-PD-1 antibodies act to eliminate the states (58). Temporal inactivation of PD-1 expression in T cells using KI mice or KI T cells may reveal its role in the deactivation of T cells and maintenance of their anti-tumor activity.

If the degron system is improved, the function of immunosuppressive molecules such as PD-1 will be suppressed only when the drug is administered, which might reduce side effects. Although it was difficult to reduce the tumor size after an initial increase, it may be possible to prevent cancer metastasis and recurrence after the initial tumor is eradicated by drug administration. In such cases, the administration of approved pharmaceuticals is preferable. In this respect, the SMASh degron system is suitable because it employs a drug such as ASV or GRV, which is already used in the treatment of human hepatitis C. In order to investigate the effectiveness of degron-tagged PD-1 in humans, it is necessary to conduct experiments using immune-deficient mice transplanted with human HSCs carrying the PD-1-based degron system.

We acknowledge the possibility of the off-target binding of NS3/4A to endogenous proteins. Toll-IL-1 receptor domain-containing adapter protein inducing IFN-β (*Ticam1*) and Mitochondrial antiviral signaling protein (*Mavs*) play an important role in innate immunity and are cleaved by the NS3/4A protease in the SMASh degron tag (59). The possibility of a previously unknown target of the NS3/4A protease cannot be excluded. Consequently, it is necessary to consider events where the aforementioned genes may participate in when employing the SMASh degron system.

In summary, we established the SMASh degron system, which can degrade endogenous PD-1 fused with a SMASh tag in a time-specific manner. Growth of MC-38 colon adenocarcinoma cells injected into KI mice was repressed upon ASV or GRV administration. Moreover, WT mice transplanted with KI BMCs after being irradiated with lethal radiation could reject MC-38 cells in the presence of GRV. At this stage, the SMASh system used in the present study is leaky; nevertheless, more precise regulation of protein expression could be achieved in future research, and the degron system could be employed for the treatment of diseases and the study of various biological mechanisms.

## MATERIALS AVAILABILITY

All materials besides B6J-S1 WT male ESCs and tissue sections are available from C.N. ESCs are available from RIKEN BRC. The tissue sections in this study are available from T.M. All requests may be addressed to the following corresponding authors:

Masahide Asano, Ph.D., Professor and Chie Naruse, Ph.D., Associate Professor

Email: asano.masahide.5u@kyoto-u.ac.jp (MA), naruse.chie.6r@kyoto-u.ac.jp (CN)

## DATA AVAILABILITY

All data generated or analyzed during this study are included in this published article (and its supplementary information files). Flow Cytometry experiments is available via https://flowrepository.org/id/, ID numbers FR-FCM-Z4N7, FR-FCM-Z5B9, FR-FCM-Z5BE, FR-FCM-Z57W and FR-FCM-Z5BD.

## Supplementary Material

zcac019_Supplemental_File
