# Supplementary material for: A degron system targeting endogenous PD-1 inhibits the growth of tumor cells in mice
Source: NAR Cancer. 2022 Jun 17;4(2):zcac019. doi: 10.1093/narcan/zcac019 (PMC9204894; doi:10.1093/narcan/zcac019)
Supplement: zcac019_Supplemental_File [file zcac019_supplemental_file.pdf]

Supplementary Data

Figures and Tables

Figure S1. Structure of the SMASh degron tag.

Figure S2. Expression vectors used for transfecting EL4 and Jurkat cells.

Figure S3. Time course of mCherry degradation and reexpression in EL4 cells.

Figure S4. Flow cytometric analyses of Jurkat cells on day 4 after treatment with asunaprevir (ASV).

Figure S5. Generation and genotyping of mCherry-SMASh knock-in mice.

Figure S6. Flow cytometric analyses of splenocytes three days after treatment with concanavalin A.

Figure S7. PD-1 expression in wild-type T cells was not changed with ASV or grazoprevir (GRV) treatment.

Figure S8. MC-38 cell growth on ASV treatment for knock-in (KI) and anti-PD-1 treatment for wild-type (WT) mice.

Figure S9. Flow cytometric analyses of the tumor microenvironment of the MC-38 cell mass on day 14 of ASV administration in KI mice.

Figure S10. Tumor-infiltrating immune cells in WT mice with asunaprevir (ASV) treatment.

Figure S11. Quantification of donor cell ratio in bone marrow chimeric mice by quantitative genomic PCR for mCherry and *Bcl2*.

Table S1. Genotypes of offspring obtained after intercrossing heterozygous knock-in mice

Table S2. Primer sequences

Table S3. Antibodies used in this study

Table S4. Combinations of antibodies and dead-cell indicator for flow cytometry

Table S5. *F* values and *P* values of ANOVA

Supplemental Figures

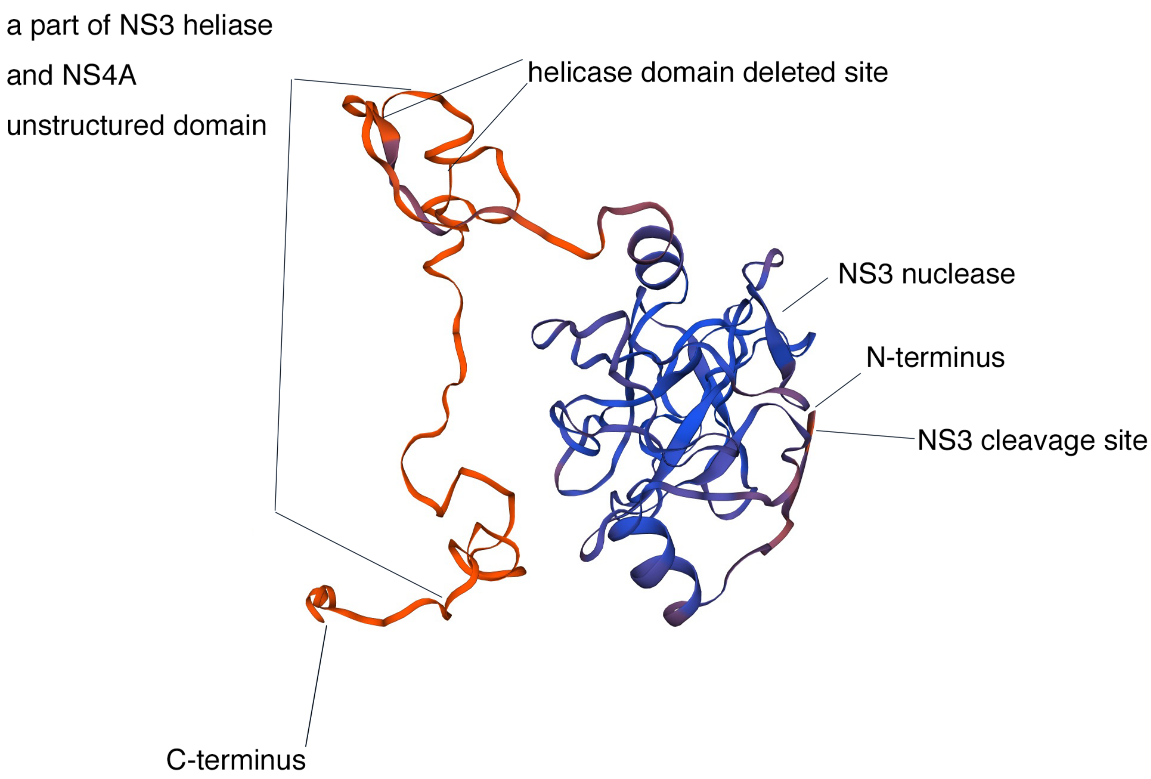

Figure S1 (related to Figure 1) Structure of the SMASh degron tag.

The structure was modeled using SWISS-MODEL (https://swissmodel.expasy.org/) using the SMASh amino acid sequence shown in Chung et al. Nature chemical biology 11 713 (2015) DOI: 10.1038/NCHEMBIO.1869. The helicase domain deleted sites are connected, but they are drawn apart by SWISS-MODEL in this figure.

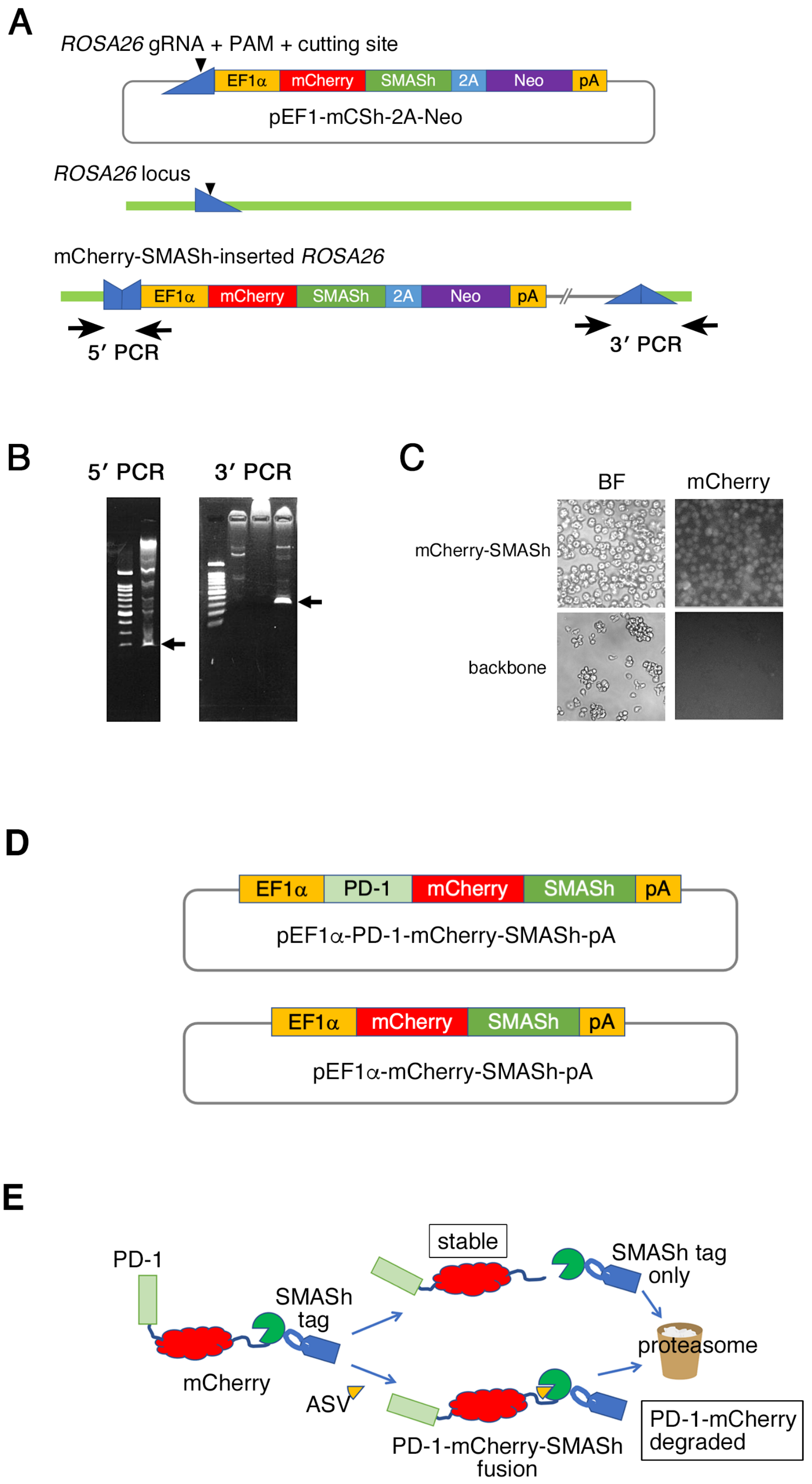

Figure S2 (related to Figure 1). Expression vectors used for transfecting EL4 and Jurkat cells.

A The *mCherry-SMASh*-expressing vector (EL4 cells; top), *ROSA26* locus (middle), and vector inserted in the *ROSA26* locus (bottom). Blue triangles indicate the guide RNA sequence. Arrowheads indicate digestion sites in Cas9-gRNA. Arrows indicate the primers used for PCR. **B** PCR for confirming the integration of *mCherry-SMASh*-expressing vector into the *ROSA26* locus. Arrows indicate the 160 bp product by 5′ PCR and 354 bp product by 3′ PCR. **C** Fluorescent mCherry (right) and bright-field (BF) images (left) after G418 selection of EL4 cells following transfection with the *mCherry-SMASh*-expressing vector (top) and backbone vector (bottom). **D** Construction of *Pdcd1-mCherry-SMASh*-expressing vector (top) and the *mCherry-SMASh*-expressing vector (Jurkat cells; bottom). **E** Schematic diagram of the SMASh degron system in the PD-1-mCherry fusion protein. EF1α, human elongation factor 1α promoter; PD-1, mouse *Pdcd1* cDNA; mCherry, monomeric derivative of DsRed fluorescent protein sequence; SMASh, Small molecule-assisted shutoff degron tag; 2A, 2A self-cleaving peptide sequence from *Thosea asigna* virus; Neo, neomycin resistance gene; pA, SV40 poly(A) additional signal; ASV, asunaprevir.

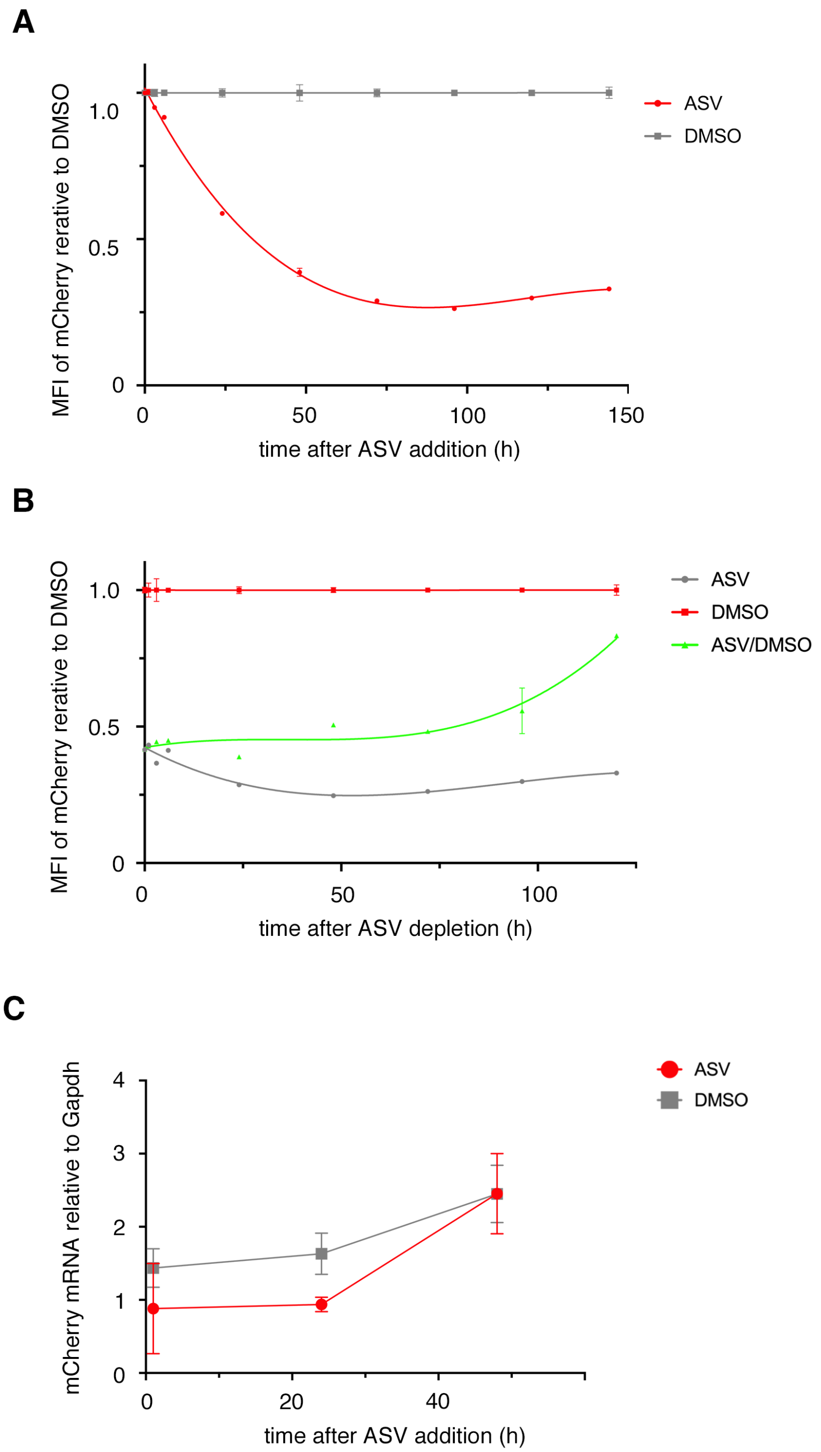

Figure S3 (related to Figure 1). The time course of the amount of mCherry protein and mRNA in mCherry-SMASh-expressing EL4 with ASV and removal of ASV.

A, B The time course of the fluorescence intensity of mCherry in mCherry-SMASh-expressing EL4 cells with 10 μM ASV treatment (**A**) and removal of ASV after 48 h-treatment with

ASV (**B**). Third-order regression curves were drawn using Prism 9. Each data was obtained from three independent samples. Data are represented as mean ± standard error (SE). Some error bars are shorter than the size of the symbols. **C** The time course of the amount of mCherry mRNA, measured by RT-qPCR. Each data was obtained from three independent samples. Data are represented as mean ± SE. Two-tailed unpaired *t*-test showed no difference between the ASV-treated group (ASV) and the untreated group (DMSO).

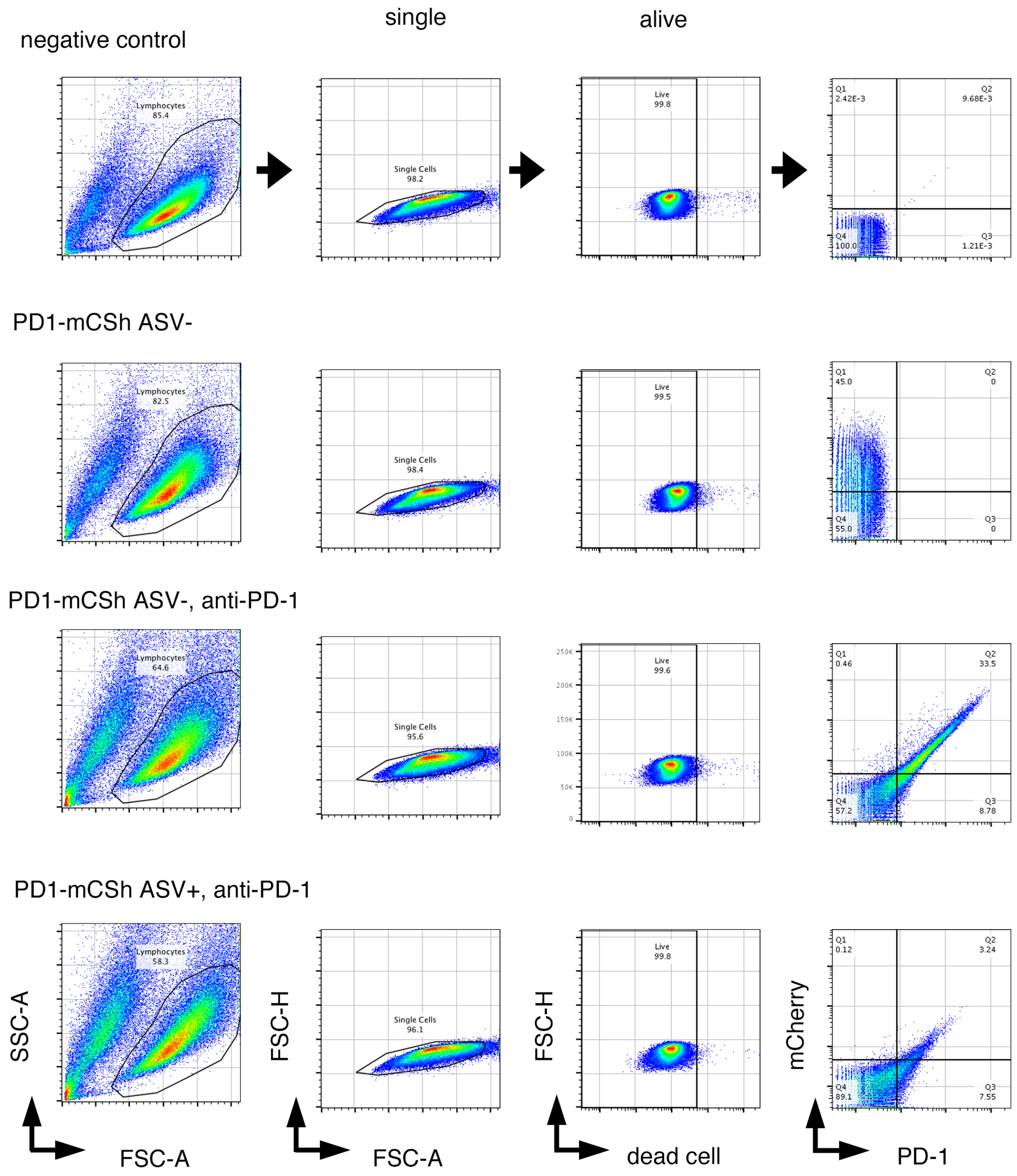

Figure S4 (related to Figure 1). Flow cytometric analyses of Jurkat cells on day 4 after treatment with asunaprevir (ASV).

Expression analysis of PD-1 and mCherry in Jurkat cells transfected with the vector backbone (negative control), PD-1-mCSh-expressing cells without ASV (PD1-mCSh ASV-), anti-PD-1-immunostained PD-1-mCSh-expressing cells without ASV (PD1-mCSh ASV-, anti-PD-1), and anti-PD-1-immunostained PD-1-mCSh-expressing cells with ASV (PD1-mCSh ASV+, anti-PD-1) using flow cytometry.

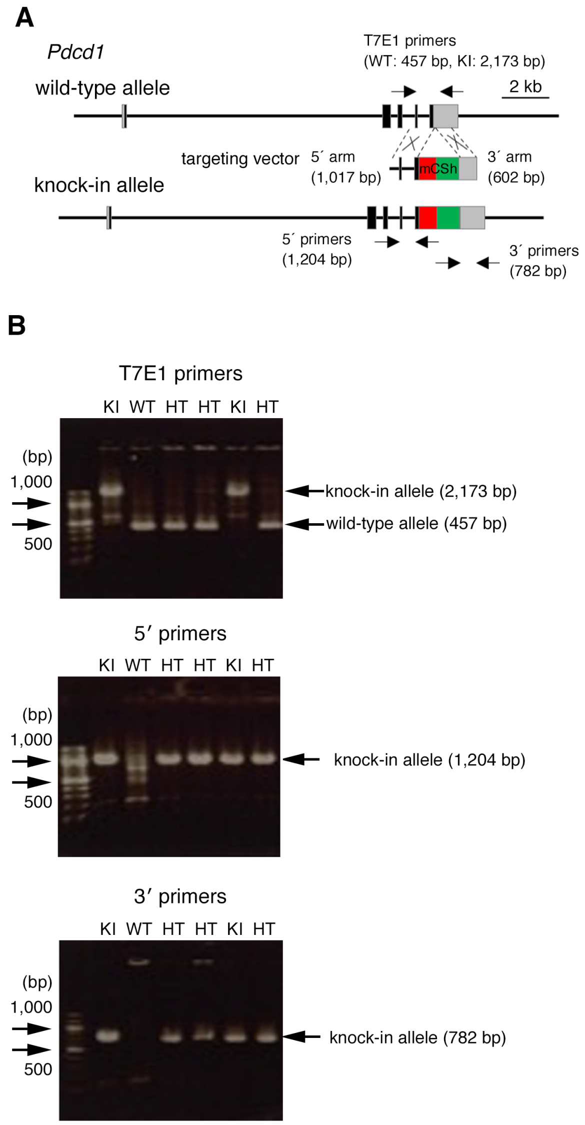

Figure S5 (related to Figure 2). Generation and genotyping of mCherry-SMASh knock-in mice.

A Strategy for insertion of mCherry-SMASh downstream of the PD-1 coding region. Black box: protein-coding region in *Pdcd1* exons, grey box: non-coding region in the last exon, red box: mCherry gene, green box: SMASh degron tag, arrows: PCR primers for genotyping. **B** Genotyping of wild-type (WT), heterozygous mutant (HT) and homozygous mutant (KI) mice by PCR using T7E1 primers, 5′ primers, and 3′ primers shown in Figure 2A. The PCR products of the knock-in and the wild-type alleles are indicated by arrows. As the PCR product of the knock-in allele (2,173 bp) obtained using the T7E1 primers was longer than that obtained for the wild-type allele (457 bp) in heterozygotes, the longer knock-in allele PCR product could not be detected owing to competition for amplification with the shorter wild-type allele PCR product.

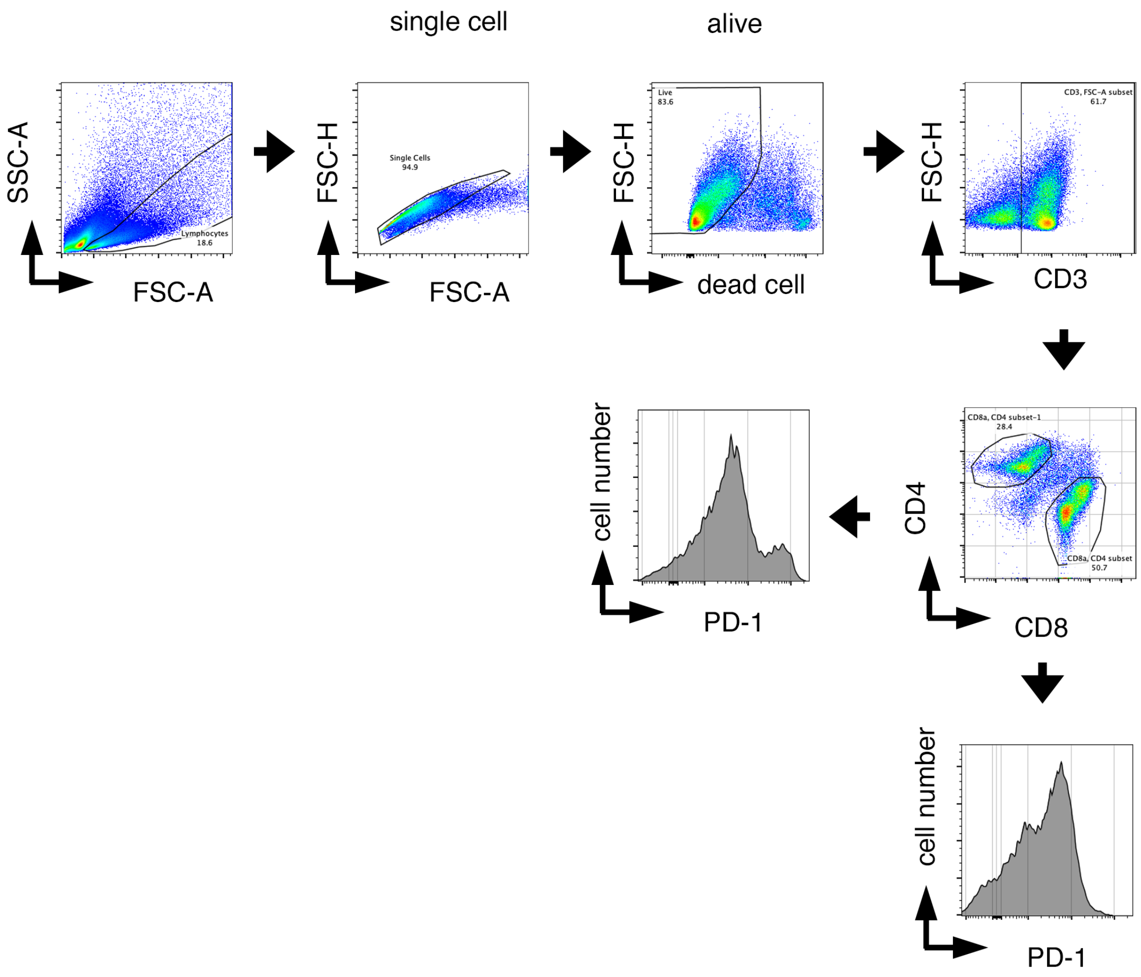

Figure S6 (related to Figure 2). Flow cytometric analyses of splenocytes three days after treatment with concanavalin A.

Analysis of PD-1 and mCherry expression in PD-1-mCherry-SMASh knock-in homozygous $CD3^+$ T cells from splenocytes by flow cytometry.

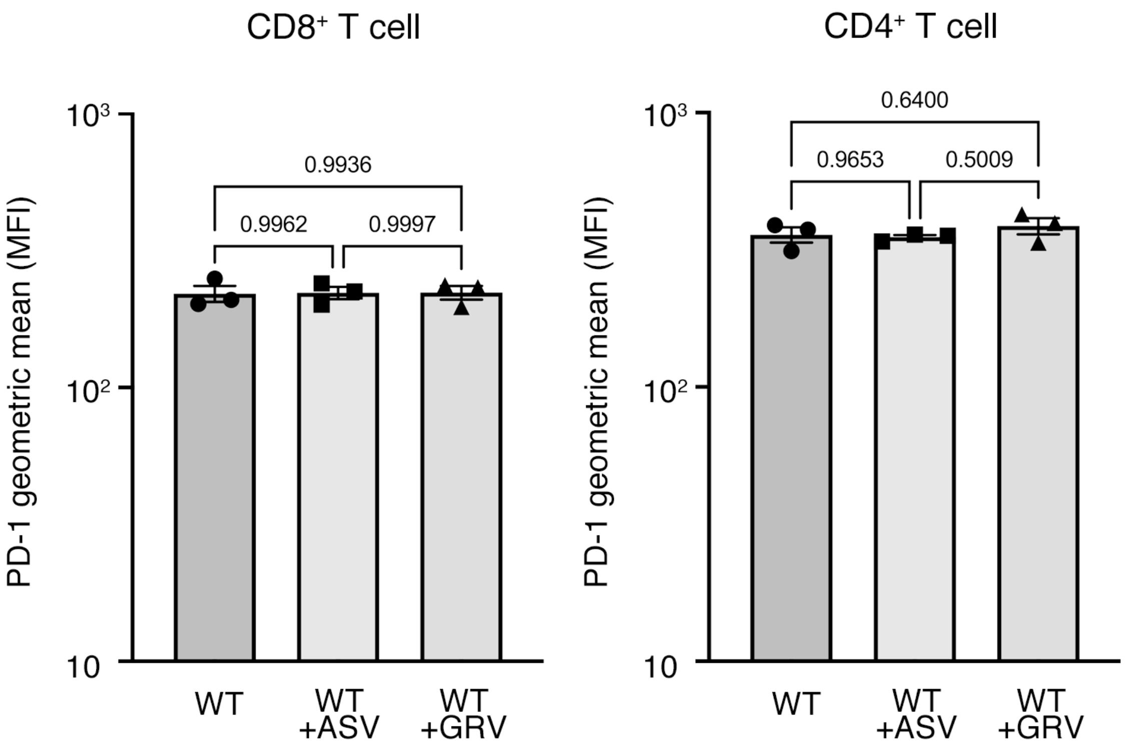

Figure S7 (related to Figure 2). PD-1 expression in wild-type T cells stimulated by concanavalin A with ASV or GRV treatment.

The geometric means of fluorescence intensity of PD-1-FITC in wild-type $CD8^+$ (left) and $CD4^+$ (right) T cells with and without ASV or GRV. The experiment was performed independently for splenocytes from three mice. The experiment was performed once. A geometric mean of each sample was calculated by subtraction of the geometric mean stained with isotype control from that stained with anti-PD-1 antibody. Data are represented as mean ± SE. *P* values obtained by one-way ANOVA were shown in the graph.

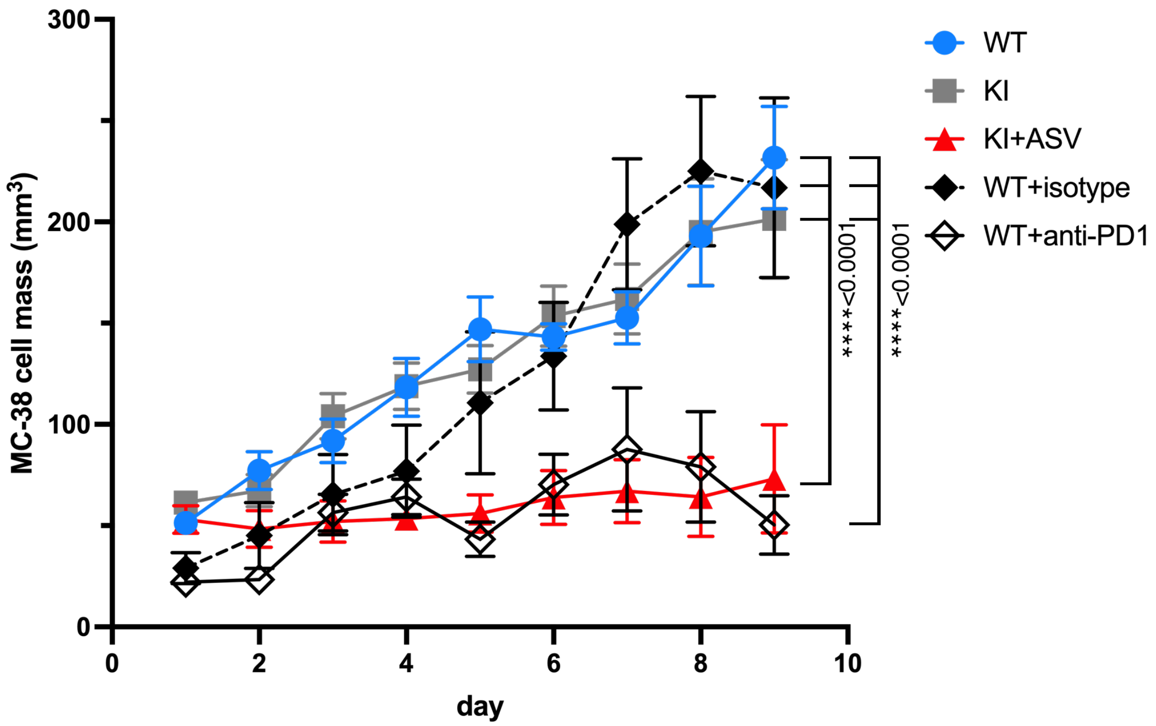

Figure S8 (related to Figure 3) MC-38 cell growth on ASV treatment for knock-in (KI) mice and anti-PD-1 treatment for wild-type (WT) mice.

Growth curve of MC-38 cells in WT with solvent (WT, n = 14), KI with solvent (KI, n = 19), KI with asunaprevir (KI+ASV, n = 15), WT with isotype control (WT+isotype, n = 6) and WT with anti-PD-1 antibody treatment (WT+anti-PD1, n = 6). One million MC-38 cells were injected subcutaneously, and MC-38 cell masses were measured after 4 days. When cell masses reached a volume greater than 45 mm^3, the administration of was initiated. The experiment for WT+isotype control and WT+anti-PD-1 antibody treatment was performed once. The experiment for WT, KI, and KI+ASV was repeated three times. The results are represented as a single graph. ****$p < 0.0001$; ordinary two-way ANOVA.

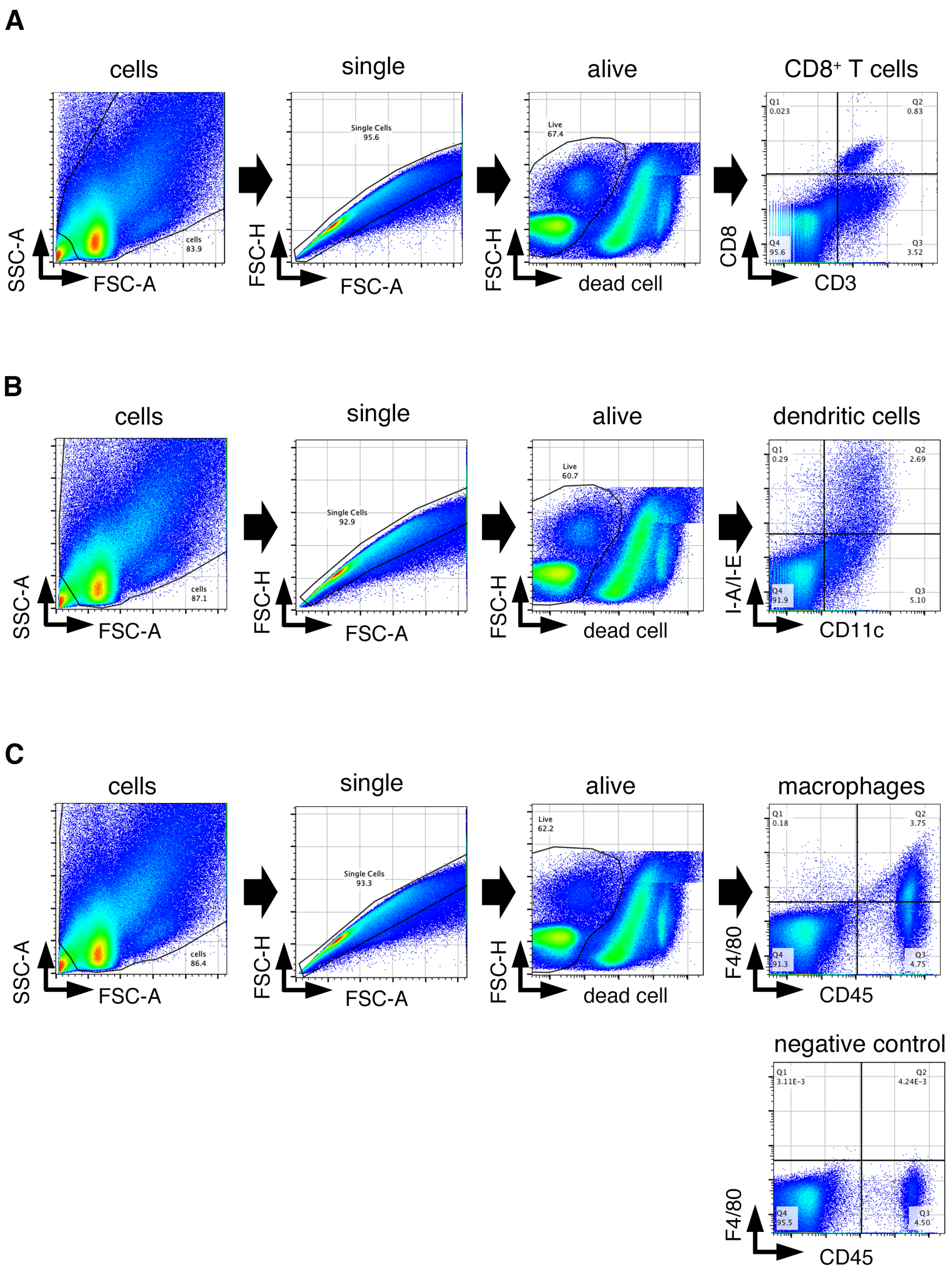

Figure S9 (related to Figure 4). Flow cytometric analyses of the tumor microenvironment of the MC-38 cell mass on day 14 of asunaprevir (ASV) administration in knock-in (KI) mice.

A Separation of $CD8^+$ T cells using anti-CD3 and anti-CD8 antibodies for expression analyses of PD-1, TIM-3, LAG-3, IFNγ, CD44, and CD103. **B** Separation of dendritic cells using anti-CD11c and anti-I-A/I-E antibodies. **C** Separation of monocytes and macrophages using anti-CD45 and anti-F4/80 antibodies. The bottom panel shows the negative control for the anti-F4/80 antibody.

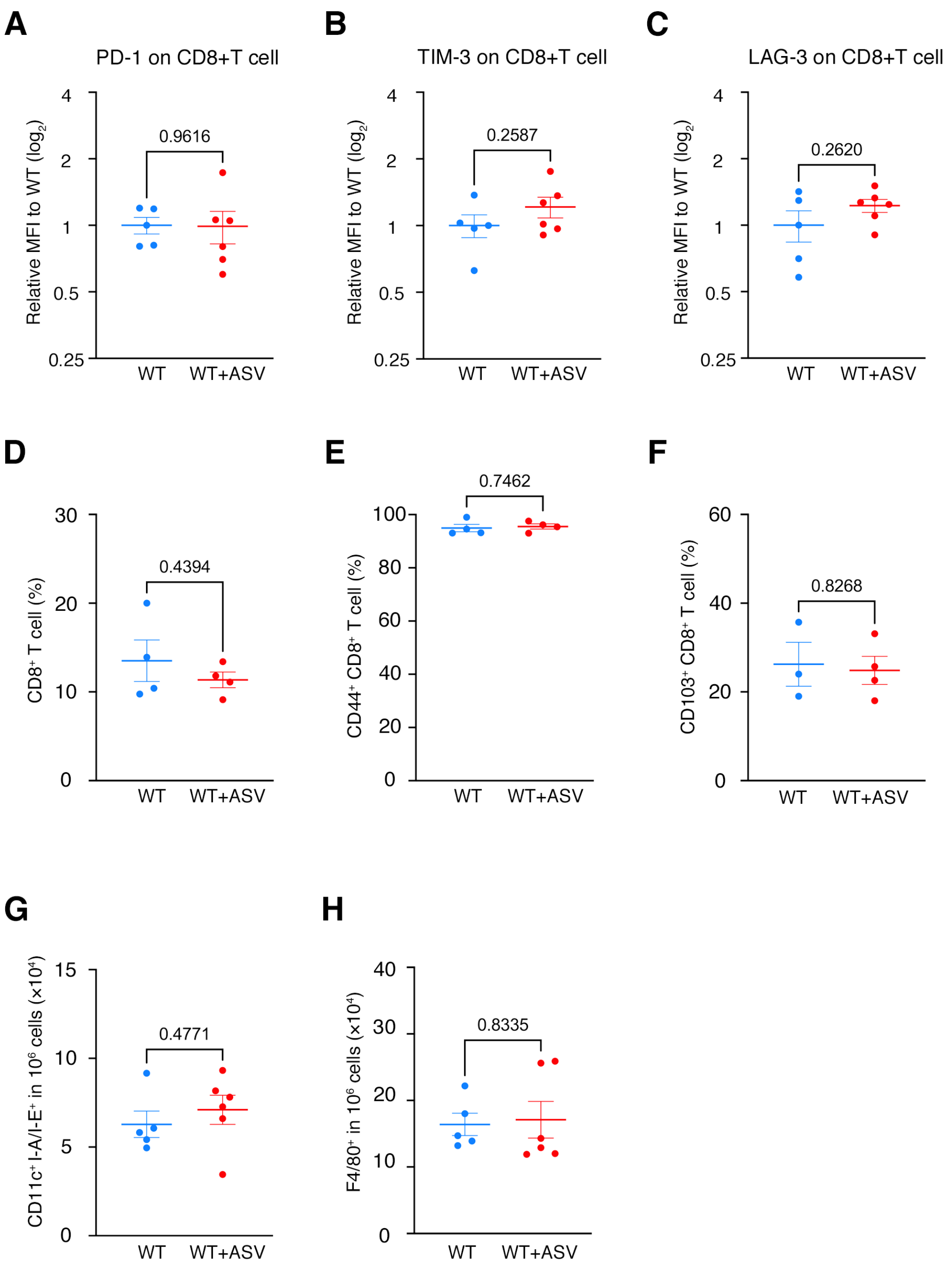

Figure S10 (related to Figure 4). Tumor-infiltrating immune cells in wild-type (WT) mice with asunaprevir (ASV) treatment.

A-**C** Cell -surface expression of PD-1 (**A**), TIM-3 (**B**), and LAG-3 (**C**) in $CD8^+$ T cells in the microenvironment of MC-38 cell masses. WT (blue, n = 5) and WT+ASV (red, n = 6), biologically independent samples. **D-F** The ratios of tumor-infiltrating $CD8^+$ T cells in CD45+ cells (**D**), $CD44^+CD8^+$ effector T cells (**E**) and $CD103^+CD8^+$ residual memory T cells (**F**) analyzed by flow cytometry. WT (blue, n = 4 in **D** and **E**, n = 3 in **F**) and WT+ASV (red, n = 4), biologically

independent samples. **G, H** The numbers of tumor-infiltrating dendritic cells (**G**) and macrophages (**H**) in 10^6 cells of microenvironment. WT (blue, n = 5) and WT+ASV (red, n = 6), biologically independent samples. Data are represented as mean ± standard error (SE). *P* values obtained by unpaired two-tailed *t*-tests are shown in the graph. The experiment was conducted once.

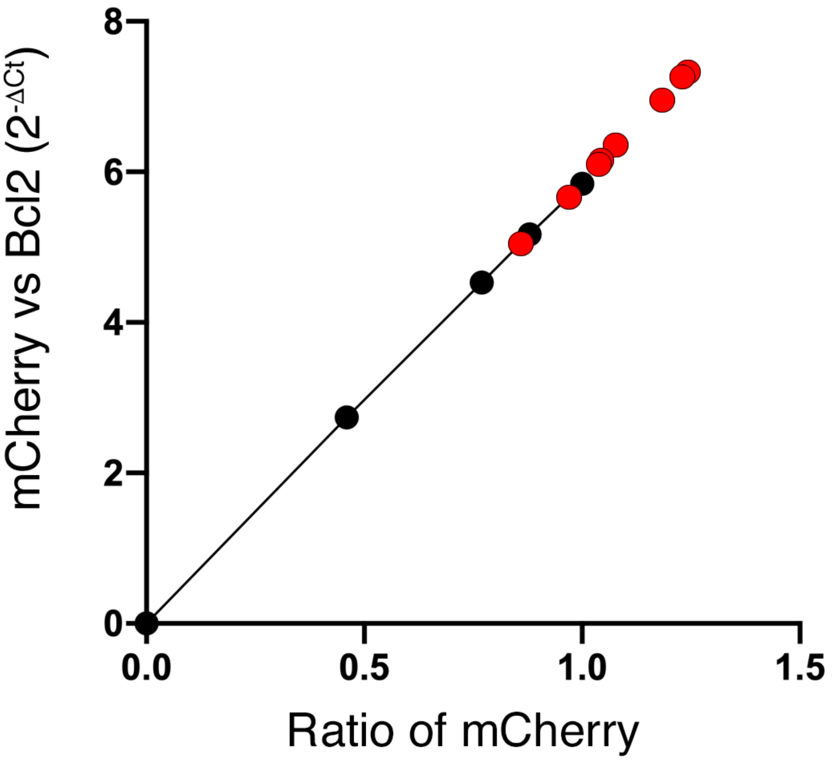

Figure S11 (related to Figure 5). Quantification of donor cell ratio in bone marrow chimeric mice by quantitative genomic PCR for mCherry and *Bcl2*.

An example of the quantification of donor cell ratio. The standard curve for calculating the ratio of $mCherry^+$ knock-in (KI) cells vs wild-type (WT) cells in peripheral blood at day 40 after transplantation of KI bone marrow cells (BMCs) into WT mice. Twenty nanograms of genomic DNA was used for quantitative PCR for *mCherry* in KI cells and the amount of *Bcl2* was used as the reference. The genomic DNA derived from the peripheral blood of WT and KI mice was adjusted so that the content of the KI genome was 0%, 46%, 77%, 88%, and 100%. The percentage of KI cells was estimated by fitting the regression line to the results of quantitative PCR using genomic DNA derived from the peripheral blood of mice transplanted with KI BMCs. Black: standard sample (KI/WT = 0, 0.46, 0.77, 0.88 and 1, respectively), red: peripheral blood from WT recipient mice transplanted with KI BMCs (n = 8). KI, knock-in; WT, wild-type; BMCs, bone marrow cells.

Supplemental Tables

Table S1. Genotypes of offspring obtained after intercrossing heterozygous knock-in mice.

Wild-type	Heterozygote	Homozygote
45 (30.2%)	70 (47.0%)	34 (22.8%)

Table S2. Primer sequences.

Primer No.	Forward (5′ to 3′)
1	CACCGCTCCAGTCTTTCTAGAAGA
2	CGAGCTCAAGCTTCGAATTCTGATGAGATGGAAGAGTGCTCTC
3	AATCCTGGCCCATTTATGGGATCGGCCATTGAACAAG
4	TCTGCTAACATGCGGTGACGTCGAGGAGAATCCTGGCCCATTTAAATTCTGCAATTCA
5	GCCTCCTGGCTTCTGAGGAC
6	CCAGGGACCGTCGTTAAACT
7	GACCATGGTGGCGACCGGTA
8	CGGTCGCCACCATGGTCGACATGTGGGTCCGGCAGGTAC
9	AACTTGTTTATTGCAGCTTATAATGGTTAC
10	AACTTGTTTATTGCAGCTTATAATGGTTAC
11	TCTTGGCCTCTTAGTGGAGGCGGAGGTGGAGTGAGCAAGGGCGAGGAGG
12	GAACGGCCACGAGTTCGAGA
13	GTCTCCTCTGACTTCAACAGCG
14	GTGCTTGCCCATGGTGTAGA
15	ATCTGACCAGATTCTTCAGCCATTAG
16	TCTTGGCCTCTTGATAGTGGAGGCGGAGGTGGA
17	CACCGCTGAAGAATCTGGTCAAAG
18	TGTTGTGGTACACCAGGAAAGG
19	GGTGTTCACGGACAACTCCT
20	GAACGGCCACGAGTTCGAGA
21	AAGCTGTCACAGAGGGGCTA
Primer No.	Reverse (5′ to 3′)
1	AAACTCTTCTAGAAAGACTGGAGC
2	TTGAATTGCAGAATTGTAGAGAACCTCCCTGTCAGGTATAATTG
3	TGAATTGCAGAATTTGGTACCTCAGAAGAACTCGTCAAGAAGGCGAT
4	TCGACGTCACCGCATGTTAGCAGACTTCCTCTGCCCTCAAATTTGTAGAGAAC
5	CCAACTTCTCGGGGACTGTG
6	AAATACTCCGAGGCGGATCA
7	GACGGACTCAGATCTCGAGCTCAA
8	CGAGATCTGAGTCCGTCGATAAGAGGCCAAGAACAATGTCCATCC
9	AAGAGGCCAAGAACAATGTCCATCCTAA
10	AGTACTAAGAGGCCAAGAACAATGTCCATCC
11	AATAAACAAGTTAGTTTAGTAGAGAACCTCCCTGTCAGGTATAATTG
12	CTTGGAGCCGTACATGAACTGAGG
13	ACCACCCTGTTGCTGTAGCCAA
14	GGACAGCAGATACGCTCACG
15	ATCAAGAGGCCAAGAACAATGTCCAT
16	AGAATCTGGTCAGATTTTGTAACCATTATAAGCTGCAATAAACAA
17	AAACCTTTGACCAGATTCTTCAGC
18	ATGATGGCCATGTTATCCTCCT
19	GGTGATGCCTGCCCTACTTA
20	CTTGGAGCCGTACATGAACTGAGG
21	CAGGCTGGAAGGAGAAGATG

Table S3. Antibodies used in this study.

Antigen	Clone	Conjugate	Company		No.	Purpose	Dilution
mCherry	Polyclonal		GeneTex	CA, US	GTX 128508	WB	1/5,000
ACTB (β-actin)	Polyclonal		Bioss	MA, US	bs-0061R	WB	1/2,000
PD-1	EPR20665		Abcam	Cambridge, UK	ab214421	WB	1/2,000
Rabbit IgG	Polyclonal	HRP	Abcam	Cambridge, UK	ab6721	WB	1/10,000
CD3	17A2	APC/Cy7	Biolegend	CA, US	100222	FCM	recommended
CD3	17A2	Pacific Blue	Biolegend	CA, US	100214	FCM	recommended
CD8a	53-6.7	AlexaFluor700	Biolegend	CA, US	100729	FCM	recommended
PD-1	29F.1A12	FITC	Biolegend	CA, US	135214	FCM	recommended
Isotype control	RTK2758	FITC	Biolegend	CA, US	400506	FCM	recommended
TIM-3	RMT3-23	PE/Cy7	Biolegend	CA, US	119715	FCM	recommended
Isotype control	RTK2758	PE/Cy7	Biolegend	CA, US	400521	FCM	recommended
LAG-3	C9B7W	PerCP/Cy5.5	Biolegend	CA, US	125211	FCM	recommended
Isotype control	RTK2071	PerCP/Cy5.5	Biolegend	CA, US	400425	FCM	recommended
CD103	2E7	PE/Cy7	Biolegend	CA, US	121425	FCM	recommended
CD44	IM7	BV421	Biolegend	CA, US	103039	FCM	recommended
CD45	30-F11	PE	Biolegend	CA, US	103105	FCM	recommended
F4/80	BM8	APC/Cy7	Biolegend	CA, US	123117	FCM	recommended
CD11c	N418	FITC	Biolegend	CA, US	117305	FCM	recommended
I-A/I-E	M5/114.15.2	PE/Cy7	Biolegend	CA, US	107615	FCM	recommended
IFNγ	XMG1.2	Pacific Blue	Biolegend	CA, US	505817	FCM	recommended
FcγR CD16/ CD32	2.4G2		TONBO	CA, US	70-0161	FCM	recommended

WB, Western blotting; FCM, flow cytometry.

Table S4. Combinations of antibodies and dead-cell indicator for flow cytometry.

CD3	17A2	Pacific Blue
CD8a	53-6.7	AlexaFluor700
PD-1	29F.1A12	FITC
TIM-3	RMT3-23	PE/Cy7
LAG-3	C9B7W	PerCP/Cy5.5
SYTOX Red Dead Cell Stain		
FcgammaR	CD16/CD32	

CD3	17A2	Pacific Blue
CD8a	53-6.7	AlexaFluor700
PD-1 isotype control	RTK2758	FITC
TIM-3 isotype control	RTK2758	PE/Cy7
LAG-3 isotype control	RTK2071	PerCP/Cy5.5
SYTOX Red Dead Cell Stain		
FcgammaR	CD16/CD32	

CD3	17A2	Pacific Blue
CD8a	53-6.7	AlexaFluor700
CD44	IM7	BV421
SYTOX Red Dead Cell Stain		
FcgammaR	CD16/CD32	

CD3	17A2	Pacific Blue
CD8a	53-6.7	AlexaFluor700
CD103	2E7	PE/Cy7
SYTOX Red Dead Cell Stain		
FcgammaR	CD16/CD32	

CD3	17A2	APC/Cy7
CD8a	53-6.7	AlexaFluor700
IFNgamma	XMG1.2	Pacific Blue
SYTOX Red Dead Cell Stain		
FcgammaR	CD16/CD32	

CD11c	N418	FITC
I-A/I-E	M5/114.15.2	PE/Cy7
SYTOX Red Dead Cell Stain		
FcgammaR	CD16/CD32	

CD45	30-F11	PE
F4/80	BM8	APC/Cy7
SYTOX Red Dead Cell Stain		
FcgammaR	CD16/CD32	

Table S5. *F* values and *P* values of ANOVA.

Figure 3C

ANOVA table	*F* (DFn, DFd)	*P* value
Time	*F* (1.944, 56.36) = 17.29	*p* < 0.0001
Column Factor	*F* (2, 27) = 10.61	*p* = 0.0004
Subject	*F* (27, 232) = 5.577	*p* < 0.0001

Figure 3F

ANOVA table	*F* (DFn, DFd)	P value
Time	*F* (1.299, 24.68) = 12.82	*p* = 0.0007
Column Factor	*F* (1, 19) = 4.493	*p* = 0.0474
Subject	*F* (19, 190) = 9.107	*p* < 0.0001

Figure 5C

ANOVA table	*F* (DFn, DFd)	P value
Time	*F* (2.125, 29.75) = 8.975	*p* = 0.0007
Column Factor	*F* (2, 14) = 10.49	*p* = 0.0016
Subject	*F* (14, 196) = 14.08	*p* < 0.0001
